# Machine Learning Model for Sepsis Prediction in Prolonged and Chronic Critical Illness: Development and Validation Using Retrospective Real-World ICU Data

**DOI:** 10.3390/jcm15020777

**Published:** 2026-01-18

**Authors:** Mikhail Ya. Yadgarov, Olga Yu. Rebrova, Levan B. Berikashvili, Petr A. Polyakov, Kristina K. Kadantseva, Alexey A. Yakovlev, Andrey V. Grechko, Valery V. Likhvantsev

**Affiliations:** 1 Federal Research and Clinical Center of Intensive Care Medicine and Rehabilitology, Moscow 107031, Russia; lberikashvili@fnkcrr.ru (L.B.B.); p.polyakov@fnkcrr.ru (P.A.P.); kkadanceva@fnkcrr.ru (K.K.K.); ayakovlev@fnkcrr.ru (A.A.Y.); avgrechko@fnkcrr.ru (A.V.G.); lik0704@gmail.com (V.V.L.); 2 Pirogov Russian National Research Medical University, Moscow 117997, Russia; o.yu.rebrova@gmail.com; 3 I.M. Sechenov First Moscow State Medical University, Moscow 119991, Russia

**Keywords:** sepsis prediction, machine learning, chronic critical illness, intensive care unit, right-aligned model, SHAP, real-world data

## Abstract

**Background:** No machine learning (ML) models for sepsis prediction have been specifically developed for patients with prolonged or chronic critical illness (PCI/CCI). **Objective:** This study aimed to develop and validate an ML-based sepsis prediction model for this cohort. **Methods:** We analyzed ICU admissions from the Russian Intensive Care Dataset (RICD, 575 patients with PCI/CCI) and two public ICU datasets from the PhysioNet (>40,000 patients with acute critical illness). Models were trained within a right-aligned prediction framework using a case–crossover–control sampling approach and a 6 h prediction window. Two strategies were evaluated: (1) a PCI/CCI-focused model trained on RICD with external testing on PhysioNet data and (2) a universal model trained on combined RICD and PhysioNet cohorts. Models were developed with tree-based algorithms (XGBoost, LightGBM, Random Forest, AdaBoost), with internal and external validation. Primary outcome was model discrimination (AUROC). Subgroup analyses were performed for sepsis phenotypes. **Results:** The PCI/CCI-focused XGBoost model achieved an AUROC of 0.82 in the RICD cohort but failed to generalize to external ICU populations (AUROC 0.47). A universal model trained on mixed data demonstrated reduced discrimination in PCI/CCI patients (AUROC mean difference 0.02, *p* = 0.0012). Respiratory rate, heart rate, body temperature, and age were among the most important features. Predictive performance was higher in hypoinflammatory sepsis phenotype (AUROC 0.84 vs. 0.81 for hyperinflammatory, *p* < 0.001). Despite worthless positive predictive value (up to 21%) for PCI/CCI-focused model, negative predictive value exceeded 97%. **Conclusions:** A right-aligned ML model tailored to PCI/CCI demonstrated strong internal performance for sepsis exclusion but limited cross-population generalizability, underscoring the need for population-specific prediction models and prospective validation before clinical application.

## 1. Introduction

Sepsis is a life-threatening condition characterized by organ dysfunction due to a dysregulated host response to infection, remaining a critical global health challenge despite advances in intensive care [1]. Recent estimates indicate that sepsis affects approximately 31.5 million individuals worldwide each year, with over 19 million cases progressing to severe sepsis and accounting for approximately 5.3 million deaths annually [2].

Timely recognition and initiation of treatment within the so-called “golden hour” have been consistently associated with improved clinical outcomes [3,4,5]. However, early detection remains a persistent challenge. Traditional clinical tools, such as the Sequential Organ Failure Assessment (SOFA), Systemic Inflammatory Response Syndrome (SIRS), and quick SOFA (qSOFA), have demonstrated limited efficacy for sepsis prediction in real-world settings, often resulting in delayed diagnosis and treatment initiation [6,7,8,9,10]. Recent evidence supports the systematic use of screening tools for early sepsis detection, with the 2021 Surviving Sepsis Campaign (SSC) guidelines highlighting the potential role of machine learning (ML) algorithms in enhancing screening accuracy [5]. ML-based models, particularly those employing real-time prediction strategies with right-aligned data structures, have emerged as promising tools for anticipating sepsis onset hours before clinical manifestation [11]. This approach supports clinical decision-making by enabling timely interventions, such as the early initiation of antibiotic or fluid therapy [11].

Over the past five years, several systematic reviews and meta-analyses have confirmed the efficacy of ML-based right-aligned sepsis prediction models, demonstrating their superiority over traditional clinical scoring systems [10,11,12]. Nevertheless, these models have been developed and validated in general intensive care unit (ICU) cohorts or general ward populations, limiting their applicability to specific patient subgroups. One such subgroup comprises patients with prolonged or chronic critical illness (PCI/CCI)—a distinct clinical condition characterized by persistent organ dysfunction, extended ICU stays, and increased risk of complications, including healthcare-associated infections and sepsis [13,14]. Despite growing clinical interest, no universally accepted definition of PCI/CCI exists to date [15], and no sepsis prediction models have been developed for this population.

The aim of this study was to develop and validate a right-aligned ML model for sepsis prediction in ICU patients with PCI/CCI using real-world data.

## 2. Materials and Methods

### 2.1. Source of Data

The Federal Research and Clinical Center of Intensive Care Medicine and Rehabilitology (FRCC ICMR) is one of the largest tertiary centers in the Russian Federation, specializing in the management and research of PCI/CCI. The primary data source for this study was the institutional Russian Intensive Care Dataset (RICD) v2.0 [16,17], an anonymized dataset comprising 8420 ICU admissions from 3404 unique patients treated at FRCC ICMR, totalling 252,836 patient-days. In addition, two publicly available datasets from the PhysioNet/Computing in Cardiology Challenge 2019 (Sepsis Prediction Challenge) [18,19,20] were used both for model development and external validation. Challenge-1 dataset contains data from 20,336 ICU patients admitted to Beth Israel Deaconess Medical Center (Boston, MA, USA), and Challenge-2 dataset includes data from 20,000 ICU patients admitted to Emory University Hospital (Atlanta, GA, USA). Unlike the RICD cohort, the Challenge datasets reflect ICU populations with acute critical conditions. Both Challenge datasets have been widely used as benchmarks for the development and validation of ML models in sepsis prediction research [21,22,23,24,25,26]. They were selected for this study due to their large size, structured time-series format, availability of sepsis onset annotations, and the absence of other publicly accessible datasets representing patients with PCI/CCI beyond the institutional RICD dataset.

All datasets provide structured hourly time-series data on vital signs, laboratory values, and demographic characteristics, with sepsis onset annotated according to the Sepsis-3 criteria [5].

### 2.2. Study Design and Setting

For the purpose of this real-world study, we screened all ICU admissions recorded in the RICD dataset between December 2017 and September 2024. Patients were eligible for inclusion if their ICU length of stay was ≥24 h and if at least one hourly sepsis assessment based on the Sepsis-3 criteria was available during their ICU stay. Patients were excluded if no data on continuously monitored vital signs or therapeutic interventions were recorded throughout the ICU admission. To avoid duplication, only the first ICU admission for each patient was included.

The external Challenge datasets from the PhysioNet/Computing in Cardiology Challenge 2019, covering ICU admissions from 2009 to 2019, were used according to their original structure and inclusion criteria, and no additional exclusion criteria were applied.

All eligible cases from the datasets covering an 8–10-year period were included; no formal sample size calculation was required. The study protocol was approved by the FRCC ICMR local ethics committee (approval No. 1/24/1, 24 April 2024). The study protocol was not prospectively registered. The study was conducted in accordance with the Transparent Reporting of a multivariable prediction model for Individual Prognosis Or Diagnosis (TRIPOD) guidelines [27,28]. The TRIPOD + AI checklist is provided in Supplemental Materials S1.

### 2.3. Data Management

Data extraction and preprocessing were performed using DB Browser for SQLite v.3.13.1 and Python v.3.12. All data processing scripts are available at GitHub (https://github.com/MikhailYadgarov/RICDv2-sql-code, accessed on 15 October 2025).

In the institutional RICD dataset, Sepsis-3 assessment was performed on a daily basis. In the absence of SOFA measurements at ICU admission, baseline SOFA was considered unavailable, and Sepsis-3 assessment was possible only if at least one SOFA score was available within the preceding two days. SOFA scores were not recalculated but were used directly as recorded in the clinical data. Suspected infection was based on the presence of microbiological cultures and/or administration of antibiotic therapy. The end of a sepsis episode was defined as the cessation of antibiotic therapy. For the PhysioNet/Computing in Cardiology Challenge 2019 datasets (Challenge-1 and Challenge-2), the original Sepsis-3 labels provided by the dataset authors were used without modification.

The initial feature set was defined using a hybrid approach, combining expert knowledge, precedent-based inclusion of features from prior sepsis prediction studies [11,12], and hand-crafted features derived from raw time-series data, which have been shown to improve model performance in sepsis prediction tasks [29]. Feature selection across vital signs, laboratory values, demographics, and comorbidities was performed using mutual information analysis, followed by a Spearman rank correlation analysis to reduce multicollinearity. Features with strong pairwise correlations (r ≥ 0.9) were excluded, favoring those with higher information gain. Final predictor importance was confirmed using SHapley Additive exPlanations (SHAP) values [30]. A complete list of features is provided in Supplemental Materials S2.

The following variables were extracted and analyzed: (1) the occurrence/absence of sepsis for all prediction windows; (2) baseline characteristics, including sex and age; (3) severity scores at ICU admission (available for RICD only); (4) laboratory parameters at admission and dynamically during the ICU stay; (5) comorbidities (not available in PhysioNet/Computing in Cardiology Challenge 2019); and (6) outcomes and complications, including all-cause mortality, ICU and hospital length of stay, septic shock (according to the sepsis-3 criteria [31]), vasopressor and/or inotrope use, mechanical ventilation, and nosocomial pneumonia (available only in RICD).

Considering sepsis heterogeneity, we classified episodes as hyperinflammatory if systemic inflammatory response syndrome (SIRS) score ≥ 2 was recorded at any time during sepsis episode, and as hypoinflammatory phenotype otherwise. No universal or consensus-based definition of sepsis phenotypes currently exists [32,33,34]. The choice of the present phenotyping approach was driven by its reproducibility and feasibility in a retrospective setting.

In all datasets, vital signs, including heart rate (HR), respiratory rate (RR), body temperature, systolic blood pressure (SBP), diastolic blood pressure (DBP), mean arterial blood pressure (MBP), and oxygen saturation (SpO_2_), were recorded hourly. For each parameter, we calculated the average (avg), minimum (min) value, maximum (max) value, and standard deviation (sd) for 1-h period (calculated only when two or more values were available), and the 3 h difference (delta_3h).

**Figure 1:**
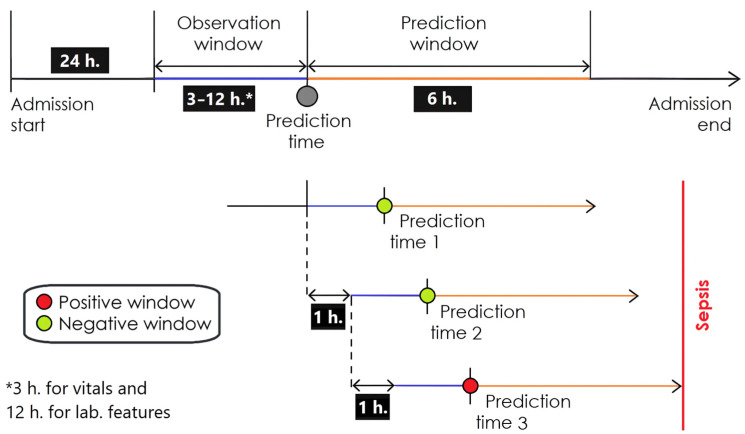
Right-aligned prediction framework with sliding time windows and defined observation and prediction windows for sepsis modeling in this study. Adapted from Lauritsen S.M. et al., npj Digital Medicine. 2021;4:158. CC BY 4.0 [32].

For the right-aligned (real-time) prediction approach, datasets were structured relative to the time of sepsis onset in patients with sepsis developed, allowing estimation of sepsis risk within a predefined prediction window [35]. Negative windows were obtained both from earlier time points in patients who later developed sepsis and from patients who did not develop sepsis, following a case–crossover–control (full-window) approach which reflect real-world conditions accurately and is currently recommended for sepsis real-time prediction model development and validation [29,36]. The models were trained to predict sepsis within the next 6 h (prediction window) based on data collected during the preceding 3 h for vital signs and 12 h for laboratory parameters (observation window), using sliding windows with a 1 h shift. This sampling approach was consistently applied across all datasets. The prediction framework is illustrated in Figure 1.

### 2.4. Statistical Analysis and Model Development

Continuous variables were summarized using medians with interquartile ranges (IQR), while categorical variables were reported as absolute numbers and percentages. Normality was assessed using the Shapiro-Wilk test. Continuous variables were compared using non-parametric tests: the Mann-Whitney U test for two groups and the Kruskal-Wallis test for three or more groups. Categorical variables were compared using the χ^2^ test or Fisher’s exact test. Two-sided *p*-values < 0.05 were considered statistically significant. Bonferroni correction was applied by multiplying *p*-values by the number of comparisons while retaining the significance threshold.

Two strategies were applied for predictive modeling. The first approach, focused on the prediction of sepsis in patients with PCI/CCI, used the RICD dataset random split into training (60%), validation (20%), and internal test (20%) subsets. The second approach aimed to develop a universal sepsis prediction model by combining 80% of RICD with the Challenge-1 dataset (acute critically ill populations), followed by an 80:20 split for training and validation, using the same 20% of RICD for internal validation as in the first approach. External validation was performed on the Challenge-2 dataset in both approaches.

All ML models—Extreme Gradient Boosting (XGBoost), Adaptive Boosting (AdaBoost), Random Forest, and LightGBM—were trained within the right-aligned prediction framework, using the same observation and prediction windows, feature engineering pipeline, and sampling strategy across all datasets. The XGBoost and LightGBM models incorporated class imbalance correction by applying the scale_pos_weight parameter, which was calculated as the ratio of negative to positive samples in the training dataset, and these weights were accounted for in the loss function during model training. For Random Forest, class imbalance was addressed using the class_weight = ‘balanced’ option, which scales class weights inversely to class prevalence. Hyperparameter optimization was performed using grid search with predefined parameters and early stopping criteria. The optimal hyperparameters were selected based on the area under the receiver operating characteristic curve (AUROC) on the validation set.

All predictors were used in their original scale without standardization or transformation, as all applied tree-based models are scale-invariant. Missing values were replaced with a constant placeholder (−999) prior to model training to ensure compatibility across all applied algorithms, including those unable to handle missing values natively (e.g., AdaBoost, Random Forest) [37]. XGBoost and LightGBM natively handle missing values by assigning them to the child node (left or right) that minimizes the loss function at each split, with the learned default direction stored in the model.

Model performance was evaluated on both internal and external test sets. The primary evaluation metric was the area under the receiver operating characteristic curve (AUROC), reported with 95% confidence intervals (CIs) and standard deviations (SDs). Optimal cut-off values were determined using Youden’s index, with additional thresholds identified based on the maximization of the positive likelihood ratio (LR+). Secondary metrics included sensitivity, specificity, positive and negative predictive values (PPV, NPV), accuracy (Acc) (adjusted for event prevalence), and F1 score. The F1 score was defined as the harmonic mean of precision and recall: F1 = 2 × (precision × recall)/(precision + recall).

Model robustness was assessed using a leave-one-patient-out (LOPO) sensitivity analysis. Comparative analysis of AUROC between models was conducted using the DeLong method [38]. In the absence of statistically significant differences between AUROC values, the highest value was selected based on simple arithmetic comparison. A predefined subgroup analysis was conducted to evaluate model performance separately in patients with hyperinflammatory and hypoinflammatory sepsis phenotypes. Decision curve analysis (DCA) was also conducted to assess the clinical utility of the model across a range of threshold probabilities.

Statistical analyses and model development were performed using IBM SPSS Statistics v. 29.0 (IBM Corp., Armonk, NY, USA) and Python (v3.12). PPV, NPV, and Acc were assessed using MedCalc web application. SHAP summary plots, calibration curves, and force plots were used to visualize and interpret model predictions.

Detailed model-building code, preprocessing scripts, and hyperparameter configurations are available on GitHub (https://github.com/MikhailYadgarov/Sepsis-prediction, accessed on 15 October 2025).

## 3. Results

### 3.1. Patient Characteristics

**Figure 2:**
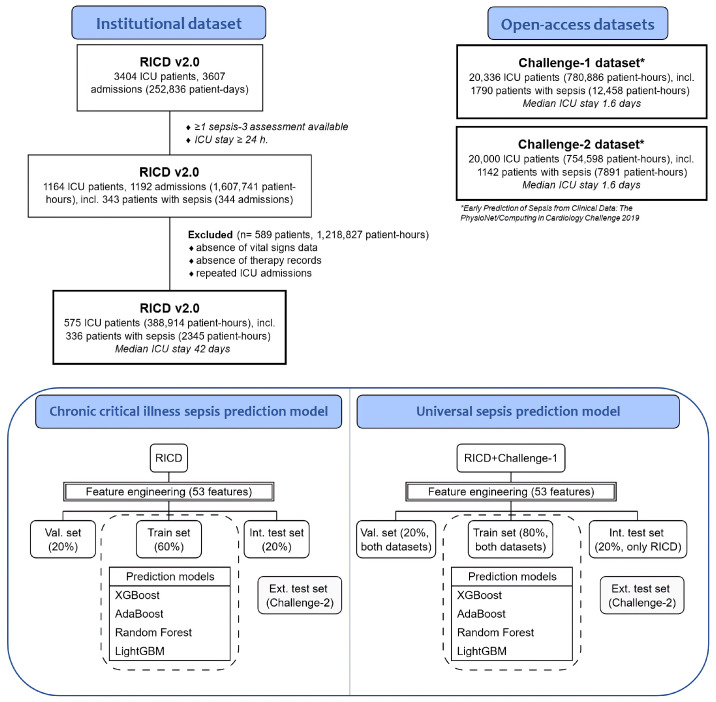
Flowchart and data allocation into training, validation, and test datasets.

A total of 575 patients (388,914 patient–hours) from the RICD dataset met the eligibility criteria, of whom 336 (57.0%) developed sepsis during their ICU stay (Figure 2). The RICD cohort was predominantly composed of patients with PCI/CCI [13], with a median ICU length of stay of 42 days (IQR 30–59); mechanical ventilation was required in over 95% patients, and more than 98% were transferred from other ICUs. Sepsis onset occurred markedly later in RICD (median 233 h) compared to Challenge datasets (35 h) (Figure S1). Moreover, the datasets differed in terms of sex and age distribution, with the RICD cohort exhibiting lower levels of leukocytes and higher platelet counts compared to the Challenge datasets. Detailed comparative data across datasets are provided in Tables S1–S5. Among septic patients in the RICD cohort, 243 (72%) episodes were classified as hyperinflammatory. Compared to the hypoinflammatory phenotype, these patients developed sepsis earlier, had higher C-reactive protein, leucocyte, fibrinogen, and procalcitonin levels on admission, and more frequently presented with septic shock and nosocomial pneumonia (Table S6).

### 3.2. ML Sepsis Prediction Models

Comparative baseline characteristics of training, validation, and test datasets are presented in Tables S7 and S8.

**Figure 3:**
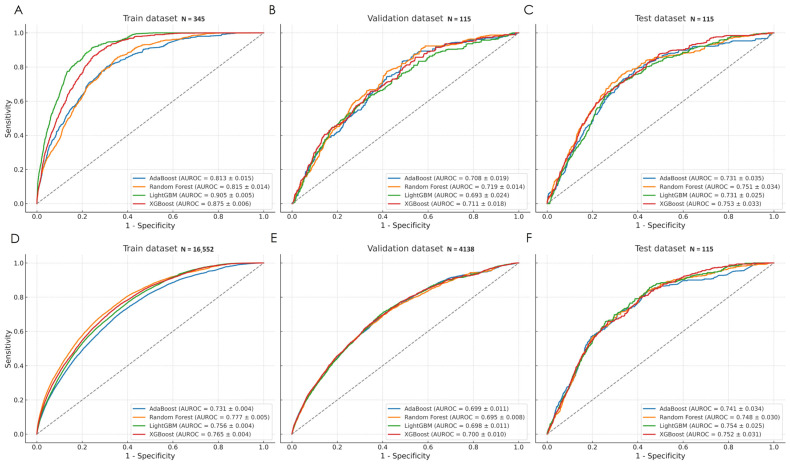
ROC-curves for ML models (sepsis prediction window 6 h). (**A**–**C**): PCI/CCI-focused approach; (**D**–**F**): universal sepsis prediction approach.

In the PCI/CCI-focused approach, the XGBoost model demonstrated the best performance, with 26 selected predictors and AUROC values of 0.875 (train), 0.711 (validation), and 0.753 (internal test) (Figure 3). Performance on the full RICD cohort reached AUROC 0.819 (Table S9). However, this model failed to generalize to external data from acute critically ill populations, yielding an AUROC of 0.474 on Challenge-2 (Figure S2). In contrast, the best-performing model in the universal approach was LightGBM, based on 25 predictors. It achieved AUROC values of 0.756 (train), 0.698 (validation), and 0.754 (internal test), with a full RICD AUROC of 0.80 and an external test AUROC of 0.655 (Figure 3, Table S9, Figure S2).

The XGBoost model from the PCI/CCI approach outperformed the LightGBM model from the universal approach for sepsis prediction in the RICD cohort (AUROC 0.819 vs. 0.802; *p* = 0.0012, Figure S3).

**Figure 4:**
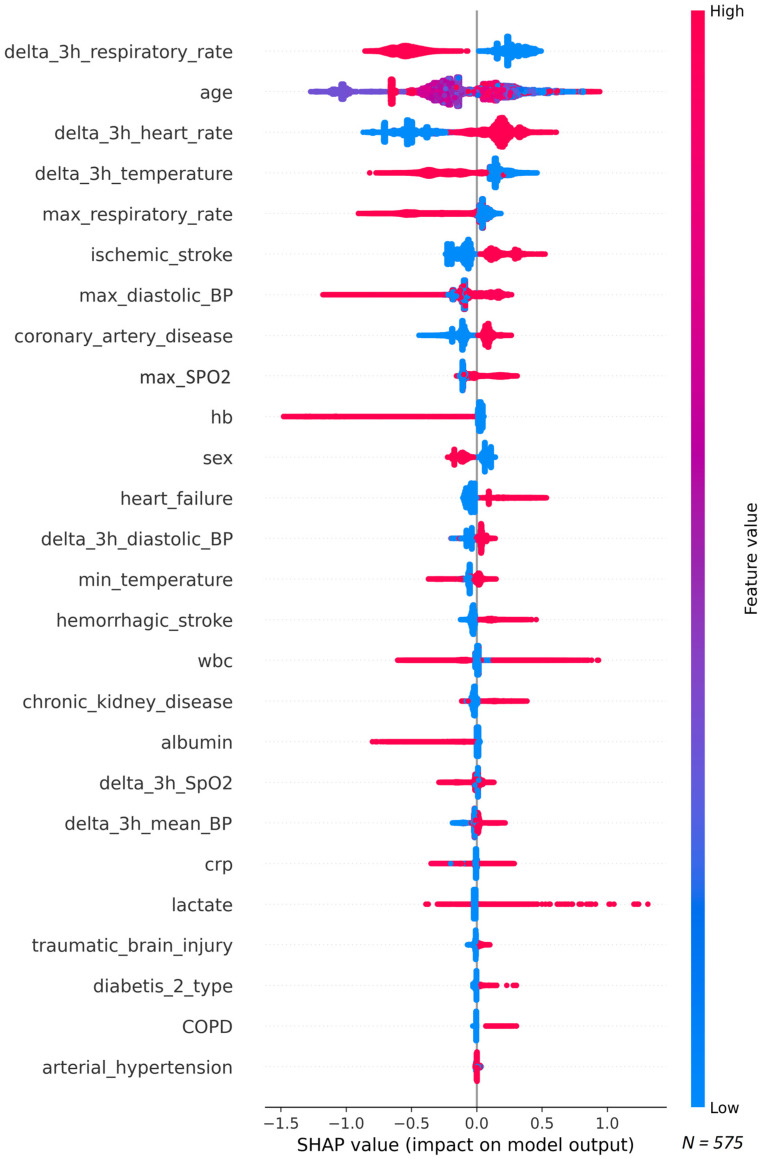
SHAP (SHapley Additive exPlanations) summary plots: impact of individual predictors on the model output.

SHAP analysis highlighted RR, age, HR, and body temperature as key contributors (Figure 4). Examples of patient-specific predictor impact are shown in Figure S4; the calibration curve is presented in Figure S5.

Multiple cut-off points were evaluated for the XGBoost model, showing high specificity (75–87%) and NPV (>97%) but low-to-moderate sensitivity (60–87%) and PPV (16–21%) across thresholds, suggesting limited reliability of positive predictions (Table S10).

Subgroup analysis revealed superior performance of the XGBoost model in patients with a hypoinflammatory phenotype (AUROC 0.843 vs. 0.810; *p* < 0.001, Figure S6), with distinct patterns of predictor contributions (Figure S7). Model robustness was confirmed by a LOPO cross-validation (SD 0.0005; 95% CI 0.818–0.820). DCA confirmed the clinical utility of the model across relevant thresholds (Figure S8). An example of predicted sepsis score dynamics is provided in Figure S9.

## 4. Discussion

### 4.1. Key Findings

In this study, we developed and validated a right-aligned ML model for early sepsis prediction using a large institutional dataset of ICU patients with PCI/CCI (RICD) and two publicly available ICU datasets. The analysis included 575 patients (388,914 patient–hours) from the RICD dataset and over 40,000 ICU patients (1,535,484 patient–hours) from the PhysioNet Challenge datasets, representing critically ill patients.

Two modeling strategies were evaluated. The PCI/CCI-focused model, trained exclusively on institutional data, demonstrated robust discrimination within this cohort (AUROC 0.819) but limited generalizability to acute critically ill populations (AUROC 0.474 in Challenge-2). In contrast, the universal model, trained on both institutional and external data, yielded more balanced performance across datasets (AUROC 0.802 in RICD; AUROC 0.655 in Challenge-2), but showed a relative loss of specificity and discrimination within the PCI/CCI subgroup.

Given these findings, further evaluation was performed using the XGBoost model derived from the PCI/CCI-focused strategy, which demonstrated the most favorable trade-off between discrimination, specificity, and alignment with the clinical characteristics of the target cohort. The final model included 26 predictors. SHAP analysis identified RR, age, HR, and body temperature as the most important features. While PPVs were low (16–21%), the model consistently achieved high specificity (up to 87%) and NPVs exceeding 97%.

Subgroup analysis showed significantly better model performance in patients with a hypoinflammatory phenotype (AUROC 0.843) compared to the hyperinflammatory phenotype (AUROC 0.810). Model robustness was supported by DCA, which demonstrated a net clinical benefit across a wide range of threshold probabilities, with a maximum net benefit of 0.237 corresponding to 24 additional correct decisions per 100 patients compared to strategies without model use. These results were further confirmed by LOPO cross-validation.

### 4.2. Relationship with Previous Studies

The findings of this study regarding the performance of sepsis prediction models are generally consistent with previously published results. Decision tree-based algorithms, including those used in our work, have demonstrated predictive performance comparable to that of neural networks, while offering better interpretability for clinical application [12]. Several studies utilizing the PhysioNet Challenge datasets reported strong predictive performance of tree-based models for sepsis prediction within a 6-h window. Chen et al. (2022) developed a random forest model achieving an AUROC of 0.850 [39]; Li et al. (2020) reported a LightGBM model with an AUROC of 0.850 [22]; Rangan et al. (2022) developed an XGBoost model with an AUROC of 0.940 [40]; and Yang et al. (2020) reported an XGBoost model with an AUROC of 0.850 [25]. However, direct comparison is limited by the absence of publicly available datasets representing PCI/CCI populations similar to RICD. Furthermore, our attempt to build a universal model applicable to both PCI/CCI and acute critically ill populations did not yield satisfactory results. This finding, together with the observation that a model with high performance in the PCI/CCI cohort failed to generalize to acute critically ill patients, can be attributed to several key factors.

First, patient characteristics in the PCI/CCI cohort differed substantially from those in the Challenge datasets. Patients with PCI/CCI had a higher incidence of sepsis and markedly longer ICU stays. Prolonged ICU stays in this population are associated with progressive anemia and a high need for transfusions, particularly in chronic critical illness [41]. Low lymphocyte and leukocyte counts are commonly observed in the course of PCI/CCI and are considered hallmarks of immune dysregulation [42]. Although elevated platelet counts are not typically associated with chronic critical illness, in our cohort, they may reflect reactive thrombocytosis secondary to systemic inflammation, likely driven by a high burden of infection—particularly pneumonia, which was documented in over 65% of patients at ICU admission.

Second, the timing and trajectory of sepsis onset differed markedly. In the RICD cohort, sepsis episodes occurred later and without a distinct peak (median 233 h), whereas in the Challenge datasets, sepsis typically developed within the first 24–48 h of ICU admission (median 35 h).

Third, the predictors of sepsis development in PCI/CCI patients differed markedly from those observed in acute critically ill populations. In acute critical care settings, sepsis is commonly associated with increased HR, RR, body temperature, and decreased blood pressure and SpO_2_ [25,40]. In contrast, in the PCI/CCI cohort, SHAP analysis identified increased HR as a shared risk factor, while lower RR, lower body temperature, higher diastolic and mean blood pressures, and higher SpO_2_ were associated with sepsis risk. Elevated blood pressure in PCI/CCI patients likely reflects the effects of vasopressor and fluid therapy administered to maintain adequate perfusion and hemodynamic stability. Lower body temperature may reflect a reduced systemic inflammatory response and impaired thermoregulation associated with immune dysfunction in chronic critical illness [43]. Decreased RR and increased SpO_2_ likely reflect the impact of mechanical ventilation. Thus, vital signs in PCI/CCI patients may serve as surrogate markers reflecting both physiological status and the effects of intensive care interventions. Other risk factors identified in our model, including patient age (although no linear association was observed) and comorbidities, are consistent with previously established predictors of sepsis [44,45,46]. Laboratory variables, including low hemoglobin, hypoalbuminemia, elevated C-reactive protein, and increased lactate levels, were also associated with sepsis risk and are supported by existing evidence [47,48,49,50]. Male sex was additionally linked to higher risk, consistent with prior reports [51,52].

Fourth, sepsis is a heterogeneous clinical syndrome characterized by marked biological and phenotypic variability. Depending on the classification approach, previous studies have identified between two and four distinct sepsis phenotypes, differing in immune response profiles, clinical course, and outcomes [32,33,34]. In our study, we stratified patients into two phenotypes based on SIRS criteria: among septic patients in the RICD cohort, 243 (72%) episodes were classified as hyperinflammatory. The predictors of sepsis onset differed between the two phenotypes in the PCI/CCI population. In the hypoinflammatory phenotype, lower CRP levels were associated with an increased risk of sepsis, likely reflecting immune suppression and insufficient inflammatory response to infection. In contrast, in the hyperinflammatory phenotype, higher CRP levels were associated with increased risk, probably indicating an exaggerated cytokine-mediated inflammatory response [53]. Similarly, the association between MBP and sepsis risk differed between groups. In the hypoinflammatory phenotype, lower MBP was associated with increased risk, possibly reflecting inadequate vascular compensatory mechanisms and evolving hypoperfusion [54]. In contrast, in the hyperinflammatory phenotype, higher MBP values may reflect the use of high-dose vasopressors required to maintain hemodynamics, and were associated with increased risk. These findings underscore the high degree of heterogeneity in sepsis and support the concept that universal prediction models are unlikely to perform adequately across different populations. Instead, prediction models should be developed with consideration of the specific demographics and clinical characteristics of the target population [55].

Fifth, differences in data structure and outcome definitions between the institutional real-world dataset and the curated PhysioNet Challenge datasets may have additionally contributed to the observed performance reduction, as the latter rely on a fixed, preprocessed feature set and predefined sepsis labels that may not fully capture the temporal complexity and clinical context of PCI/CCI populations.

The PPVs obtained at optimal cut-off points remained worthless (up to 21%), meaning that for every 100 model-generated alerts, only approximately 21 would correspond to true sepsis cases, while the remaining positive predictions would represent false positives. Low PPV is a well-known limitation of predictive models, particularly when applied to clinical scenarios with a sepsis prevalence below 50%, which may increase the rate of false alerts and limit their clinical applicability [56]. Notably, even models tested under prospective-like conditions have demonstrated similarly modest PPV values. For example, in the study by Yu et al. (2022), an ML model predicting sepsis six hours before onset achieved a PPV of 29.1% in a pseudo-prospective evaluation conducted in a general ward setting [57]. These findings highlight the need for careful interpretation of positive alerts in clinical practice. In the ICU environment, where clinicians are already exposed to a high burden of physiologic and device-related alarms, additional predictive alerts without a clearly defined action pathway may exacerbate alarm fatigue and reduce trust in decision support systems [58]. Prior studies have shown that alerts lacking clear actionability or evidence-backed response protocols are frequently ignored or overridden, limiting their potential clinical impact [59,60,61,62]. Therefore, future implementation of our sepsis prediction model must be preceded by prospective validation and explicitly address alert thresholding, expected alert burden, and integration with predefined clinical workflows to ensure that alerts prompt meaningful clinical actions.

### 4.3. Significance of the Study Findings

Our findings confirm the feasibility of developing a clinically applicable ML model for early sepsis prediction in ICU patients with PCI/CCI, a population defined by high clinical complexity, unique risk factors, and extended ICU stays.

The applicability of right-aligned prediction models has been supported by multiple studies, which demonstrated that clinical validation and subsequent integration of such models into electronic health records as components of decision support tools can facilitate earlier initiation of antimicrobial therapy and improve patient outcomes, including reduced hospital length of stay and decreased mortality rates in patients with sepsis [11,63,64,65,66].

Despite the potential advantages of ML-based predictive models, their implementation in real-world clinical practice is often challenged by concerns over algorithm transparency and interpretability, frequently referred to as the “black box” phenomenon [67]. In our study, the use of SHAP summary plots for model interpretation and SHAP force plots for individual patient-level explanations provided transparency regarding the contribution of each predictor to the model’s output. This approach may enhance clinician trust in ML-driven tools and support their acceptance in critical care settings [68].

In addition, our findings underscore the importance of population-specific model development. The significant heterogeneity in patient characteristics, sepsis trajectories, and predictor relevance observed between PCI/CCI patients and acute critically ill populations suggests that predictive models trained on generalized cohorts may underperform when applied to specialized subgroups. Consequently, tailored models, calibrated to the clinical and physiological features of target populations, are likely to offer superior clinical utility and reliability. At the current stage, the proposed model should be viewed neither as a diagnostic instrument nor as a tool for autonomous clinical decision-making or treatment initiation. Its practical role is limited to exploratory risk stratification in selected ICU populations, and use outside this context—particularly in patients with established sepsis or ongoing antibiotic therapy—may result in misinterpretation and inappropriate clinical reliance. Following successful prospective validation, the model could theoretically be used as a clinical decision support tool to assist in decisions regarding initiation or modification of antibiotic therapy, with a pragmatic threshold range of approximately 0.4–0.7, which demonstrated the greatest net clinical benefit in DCA.

### 4.4. Strengths and Limitations

To our knowledge, this is the first study to develop and validate an ML-based sepsis prediction model specifically tailored for ICU patients with PCI/CCI. To account for potential heterogeneity, the study included diverse ICU cohorts from institutional and public datasets. The case–crossover–control sampling approach (also known as the full-window strategy) used in our study has been recognized as one of the most clinically relevant methodologies for model development, providing sampling conditions closest to real-life clinical application [29,36]. Another strength is the use of external tests, still rarely performed in AI-based ICU models (reported in only 14.7% of studies [69]). Nevertheless, a full external test in PCI/CCI cohorts was not feasible due to the absence of comparable publicly available datasets representing this patient group.

Nevertheless, several limitations should be considered when interpreting our results.

First, the model was developed on a single-center cohort of PCI/CCI patients, which may affect its generalizability, although an external test was performed on multicenter datasets of acute critically ill populations. Moreover, the prolonged ICU stays, frequent inter-hospital transfers, prolonged immobilization, and delayed ICU liberation in our cohort differ from care pathways in other centers, potentially affecting model transportability to settings with shorter ICU trajectories. Second, only the first sepsis episode per patient was analyzed; recurrent episodes and their timing were not assessed. Third, although an external test was performed, the models have not been prospectively tested in clinical practice; therefore, the reported retrospective performance should be interpreted as hypothesis-generating, and prospective, outcome-based evaluation is required before any clinical deployment. Fourth, the analysis was limited to a 6-h prediction window, and the applicability of the model for longer prediction horizons remains unexplored. Fifth, two of the four applied ML algorithms (AdaBoost and Random Forest) lack native mechanisms for handling missing values, necessitating constant-value imputation, which is not an optimal strategy and may introduce bias. In the ICU setting, missingness may be informative, and our approach may have influenced both model learning and SHAP-based interpretation; no sensitivity analysis with alternative imputation strategies was performed. Sixth, the occurrence of in-hospital complications and the potential impact of therapeutic interventions were not accounted for in the prognostic model. Seventh, SHAP was used as a post-hoc attribution tool to visualize model behavior; however, SHAP values may be unstable under predictor collinearity, do not imply causality, and should not be interpreted as clinically actionable or definitive explanations of underlying pathophysiology [70,71]. Moreover, the SIRS-based sepsis phenotype stratification was used solely as an exploratory tool to evaluate heterogeneity of model performance and was not intended to redefine sepsis phenotypes, which remain an area without consensus definitions. Finally, the model demonstrated relatively low PPV and a high false-positive rate, which may affect clinical acceptance and requires further evaluation in future studies.

### 4.5. Future Studies and Prospects

The findings of this study highlight several directions for future research. First, the development and open availability of dedicated PCI/CCI datasets is essential to facilitate external validation, reproducibility, and benchmarking of predictive models in this population. Second, prospective clinical trials are warranted to assess the real-world effectiveness and clinical impact of sepsis prediction models in PCI/CCI settings. Third, the absence of a standardized definition for PCI/CCI remains a major barrier to model generalizability and cross-study comparison.

## 5. Conclusions

In this study, a right-aligned machine learning model was developed and validated for early sepsis prediction in ICU patients with prolonged or chronic critical illness, demonstrating robust discrimination within this population but limited generalizability to acute critically ill patients. The model’s performance underscores the critical importance of population-specific prediction strategies and highlights the need for tailored approaches in heterogeneous ICU populations. Further prospective studies and the development of dedicated datasets are warranted to validate these findings and to facilitate the integration of sepsis prediction models into clinical practice.

## Data Availability

Publicly and partially available datasets were analyzed in this study. The PhysioNet/Computing in Cardiology Challenge 2019 dataset is available at https://physionet.org/content/challenge-2019/1.0.0/ (accessed on 8 August 2025). The RICD dataset can be obtained upon request at https://fnkcrr-database.ru/ (accessed on 8 August 2025). Source code available at GitHub (https://github.com/MikhailYadgarov/RICDv2-sql-code (accessed on 8 August 2025); https://github.com/MikhailYadgarov/Sepsis-prediction (accessed on 8 August 2025)) and upon reasonable request.

## Supplementary Materials

The following supporting information can be downloaded at: https://www.mdpi.com/article/10.3390/jcm15020777/s1, Supplemental Materials S1. TRIPOD + AI checklist; Supplemental Materials S2. List of features; Table S1. Comparative characteristics of the three datasets; Table S2. Detailed characteristics of patients (RICD dataset); Table S3. Characteristics of vital and lab parameters (RICD dataset); Table S4. Characteristics of vital and lab parameters (Challenge-1 dataset); Table S5. Characteristics of vital and lab parameters (Challenge-2 dataset); Table S6. Comparative characteristics of patients with hypo- and hyperinflammatory sepsis phenotypes (RICD dataset); Table S7. Comparative baseline characteristics of the train set, validation set, internal and external test sets (PCI/CCI sepsis prediction model); Table S8. Comparative baseline characteristics of the train set, validation set, internal and external test sets (Universal sepsis prediction model); Table S9. AUROC values (and 95% CIs) of machine learning models for 6-h sepsis prediction across training, validation, and test sets; Table S10. Performance characteristics of best machine learning model for 6-h sepsis prediction (RICD dataset); Figure S1. Distribution of time to sepsis onset after ICU admission in three datasets; Figure S2. ROC curves of the best-performing machine learning models for early sepsis prediction (6-h window, external validation); Figure S3. ROC curves of the best-performing machine learning models for early sepsis prediction (6-h window, RICD dataset); Figure S4. Force plot illustrating predictor contributions for two patients from the RICD dataset; Figure S5. Calibration curve for XGBoost model (train set); Figure S6. ROC curves of the XGBoost model for hyperinflammatory and hypoinflammatory sepsis phenotypes (RICD dataset); Figure S7. SHAP summary plots of the XGBoost model for hyperinflammatory and hypoinflammatory sepsis phenotypes (RICD dataset); Figure S8. Decision curve analysis for the XGBoost model (RICD dataset, balanced 1:1 sample); Figure S9. Example of sepsis score dynamics over time predicted by the XGBoost model (RICD dataset, individual patient trajectory).

## Abbreviations

The following abbreviations are used in this manuscript:

Acc	Accuracy
AUROC	area under the receiver operating characteristic curve
CCI	chronic critical illness
CI	confidence interval
CRP	C-reactive protein
DCA	decision curve analysis
DBP	diastolic blood pressure
FRCC ICMR	Federal Research and Clinical Center of Intensive Care Medicine and Rehabilitology
HR	heart rate
ICU	intensive care unit
IQR	interquartile range
LOPO	leave-one-patient-out
MBP	mean blood pressure
ML	machine learning
NB	net benefit
NPV	negative predictive value
PCI	prolonged critical illness
PPV	positive predictive value
RICD	Russian Intensive Care Dataset
ROC	receiver operating characteristic
RR	respiratory rate
SBP	systolic blood pressure
SD	standard deviation
SHAP	SHapley Additive exPlanations
SIRS	systemic inflammatory response syndrome
SOFA	Sequential Organ Failure Assessment
SpO_2_	oxygen saturation
SSC	Surviving Sepsis Campaign
TRIPOD	Transparent Reporting of a multivariable prediction model for Individual Prognosis or Diagnosis
