# Supplementary material for: Machine Learning Model for Sepsis Prediction in Prolonged and Chronic Critical Illness: Development and Validation Using Retrospective Real-World ICU Data
Source: J Clin Med. 2026 Jan 18;15(2):777. doi: 10.3390/jcm15020777 (PMC12841784; doi:10.3390/jcm15020777)
Supplement: Supplementary file 1 [file jcm-15-00777-s001.zip › jcm-4075934-supplementary.pdf]

Supplementary Information

MACHINE LEARNING MODEL FOR SEPSIS PREDICTION IN PROLONGED AND CHRONIC CRITICAL ILLNESS: DEVELOPMENT AND VALIDATION USING REAL-WORLD ICU DATA

Index

Supplemental Materials S1. TRIPOD + AI checklist. 2

Supplemental Materials S2. List of features. 4

Table S1. Comparative characteristics of the three datasets. 5

Table S2. Detailed characteristics of patients (RICD dataset). 6

Table S3. Characteristics of vital and lab parameters (RICD dataset). 7

Table S4. Characteristics of vital and lab parameters (Challenge-1 dataset). 8

Table S5. Characteristics of vital and lab parameters (Challenge-2 dataset). 9

Table S6. Comparative characteristics of patients with hypo- and hyperinflammatory sepsis phenotypes (RICD dataset). 10

Table S7. Comparative baseline characteristics of the train set, validation set, internal and external test sets (PCI/CCI sepsis prediction model). 11

Table S8. Comparative baseline characteristics of the train set, validation set, internal and external test sets (Universal sepsis prediction model). 12

Table S9. AUROC values (and 95% CIs) of machine learning models for 6-hour sepsis prediction across training, validation, and test sets. 13

Table S10. Performance characteristics of best machine learning model for 6-hour sepsis prediction (RICD dataset). 14

Figure S1. Distribution of time to sepsis onset after ICU admission in three datasets. 15

Figure S2. ROC curves of the best-performing machine learning models for early sepsis prediction (6-hour window, external validation). 16

Figure S3. ROC curves of the best-performing machine learning models for early sepsis prediction (6-hour window, RICD dataset). 17

Figure S4. Force plot illustrating predictor contributions for two patients from the RICD dataset. 18

Figure S5. Calibration curve for XGBoost model (train set). 19

Figure S6. ROC curves of the XGBoost model for hyperinflammatory and hypoinflammatory sepsis phenotypes (RICD dataset). 20

Figure S7. SHAP summary plots of the XGBoost model for hyperinflammatory and hypoinflammatory sepsis phenotypes (RICD dataset). 21

Figure S8. Decision curve analysis for the XGBoost model (RICD dataset, balanced 1:1 sample). 22

Figure S9. Example of sepsis score dynamics over time predicted by the XGBoost model (RICD dataset, individual patient trajectory). 23

Supplemental Materials S1. TRIPOD + AI checklist.

Section/Topic	Item	Development / evaluation[1]	Checklist item	Reported on page
**TITLE**				
Title	1	D;E	Identify the study as developing or evaluating the performance of a multivariable prediction model, the target population, and the outcome to be predicted	1
**ABSTRACT**				
Abstract	2	D;E	See TRIPOD+AI for Abstracts checklist	1
**INTRODUCTION**				
Background	3a	D;E	Explain the healthcare context (including whether diagnostic or prognostic) and rationale for developing or evaluating the prediction model, including references to existing models	1-2
	3b	D;E	Describe the target population and the intended purpose of the prediction model in the context of the care pathway, including its intended users (e.g., healthcare professionals, patients, public)	1-2
	3c	D;E	Describe any known health inequalities between sociodemographic groups	2
Objectives	4	D;E	Specify the study objectives, including whether the study describes the development or validation of a prediction model (or both)	2
**METHODS**				
Data	5a	D;E	Describe the sources of data separately for the development and evaluation datasets (e.g., randomised trial, cohort, routine care or registry data), the rationale for using these data, and representativeness of the data	2
	5b	D;E	Specify the dates of the collected participant data, including start and end of participant accrual; and, if applicable, end of follow-up	2
Participants	6a	D;E	Specify key elements of the study setting (e.g., primary care, secondary care, general population) including the number and location of centres	3
	6b	D;E	Describe the eligibility criteria for study participants	3
	6c	D;E	Give details of any treatments received, and how they were handled during model development or evaluation, if relevant	3
Data preparation	7	D;E	Describe any data pre-processing and quality checking, including whether this was similar across relevant sociodemographic groups	3-4
Outcome	8a	D;E	Clearly define the outcome that is being predicted and the time horizon, including how and when assessed, the rationale for choosing this outcome, and whether the method of outcome assessment is consistent across sociodemographic groups	3-4
	8b	D;E	If outcome assessment requires subjective interpretation, describe the qualifications and demographic characteristics of the outcome assessors	3-4
	8c	D;E	Report any actions to blind assessment of the outcome to be predicted	3-4
Predictors	9a	D	Describe the choice of initial predictors (e.g., literature, previous models, all available predictors) and any pre-selection of predictors before model building	3-5
	9b	D;E	Clearly define all predictors, including how and when they were measured (and any actions to blind assessment of predictors for the outcome and other predictors)	3-5
	9c	D;E	If predictor measurement requires subjective interpretation, describe the qualifications and demographic characteristics of the predictor assessors	3-5
Sample size	10	D;E	Explain how the study size was arrived at (separately for development and evaluation), and justify that the study size was sufficient to answer the research question. Include details of any sample size calculation	3
Missing data	11	D;E	Describe how missing data were handled. Provide reasons for omitting any data	5
Analytical methods	12a	D	Describe how the data were used (e.g., for development and evaluation of model performance) in the analysis, including whether the data were partitioned, considering any sample size requirements	3-5
	12b	D	Depending on the type of model, describe how predictors were handled in the analyses (functional form, rescaling, transformation, or any standardisation).	5
	12c	D	Specify the type of model, rationale[2], all model-building steps, including any hyperparameter tuning, and method for internal validation	4-5
	12d	D;E	Describe if and how any heterogeneity in estimates of model parameter values and model performance was handled and quantified across clusters (e.g., hospitals, countries). See TRIPOD-Cluster for additional considerations[3]	4-5
	12e	D;E	Specify all measures and plots used (and their rationale) to evaluate model performance (e.g., discrimination, calibration, clinical utility) and, if relevant, to compare multiple models	4-5
	12f	E	Describe any model updating (e.g., recalibration) arising from the model evaluation, either overall or for particular sociodemographic groups or settings	4-5
	12g	E	For model evaluation, describe how the model predictions were calculated (e.g., formula, code, object, application programming interface)	4-5
Class imbalance	13	D;E	If class imbalance methods were used, state why and how this was done, and any subsequent methods to recalibrate the model or the model predictions	4-5
Fairness	14	D;E	Describe any approaches that were used to address model fairness and their rationale	5
Model output	15	D	Specify the output of the prediction model (e.g., probabilities, classification). Provide details and rationale for any classification and how the thresholds were identified	5

[1] D=items relevant only to the development of a prediction model; E=items relating solely to the evaluation of a prediction model; D;E=items applicable to both the development and evaluation of a prediction model
[2] Separately for all model building approaches.
[3] TRIPOD-Cluster is a checklist of reporting recommendations for studies developing or validating models that explicitly account for clustering or explore heterogeneity in model performance (eg, at different hospitals or centres). Debray et al, BMJ 2023; 380: e071018 [DOI: 10.1136/bmj-2022-071018]

Training versus evaluation	16	D;E	Identify any differences between the development and evaluation data in healthcare setting, eligibility criteria, outcome, and predictors	5
Ethical approval	17	D;E	Name the institutional research board or ethics committee that approved the study and describe the participant-informed consent or the ethics committee waiver of informed consent	3
**OPEN SCIENCE**				
Funding	18a	D;E	Give the source of funding and the role of the funders for the present study	12
Conflicts of interest	18b	D;E	Declare any conflicts of interest and financial disclosures for all authors	12
Protocol	18c	D;E	Indicate where the study protocol can be accessed or state that a protocol was not prepared	3
Registration	18d	D;E	Provide registration information for the study, including register name and registration number, or state that the study was not registered	3
Data sharing	18e	D;E	Provide details of the availability of the study data	3
Code sharing	18f	D;E	Provide details of the availability of the analytical code[4]	3, 5
**PATIENT & PUBLIC INVOLVEMENT**				
Patient & Public Involvement	19	D;E	Provide details of any patient and public involvement during the design, conduct, reporting, interpretation, or dissemination of the study or state no involvement.	NA
**RESULTS**				
Participants	20a	D;E	Describe the flow of participants through the study, including the number of participants with and without the outcome and, if applicable, a summary of the follow-up time. A diagram may be helpful.	6
	20b	D;E	Report the characteristics overall and, where applicable, for each data source or setting, including the key dates, key predictors (including demographics), treatments received, sample size, number of outcome events, follow-up time, and amount of missing data. A table may be helpful. Report any differences across key demographic groups.	Supplement
	20c	E	For model evaluation, show a comparison with the development data of the distribution of important predictors (demographics, predictors, and outcome).	Supplement
Model development	21	D;E	Specify the number of participants and outcome events in each analysis (e.g., for model development, hyperparameter tuning, model evaluation)	Supplement
Model specification	22	D	Provide details of the full prediction model (e.g., formula, code, object, application programming interface) to allow predictions in new individuals and to enable third-party evaluation and implementation, including any restrictions to access or re-use (e.g., freely available, proprietary)[5]	Supplement
Model performance	23a	D;E	Report model performance estimates with confidence intervals, including for any key subgroups (e.g., sociodemographic). Consider plots to aid presentation.	6-7, Supplement
	23b	D;E	If examined, report results of any heterogeneity in model performance across clusters. See TRIPOD Cluster for additional details[3].	Supplement
Model updating	24	E	Report the results from any model updating, including the updated model and subsequent performance	Supplement
**DISCUSSION**				
Interpretation	25	D;E	Give an overall interpretation of the main results, including issues of fairness in the context of the objectives and previous studies	8-10
Limitations	26	D;E	Discuss any limitations of the study (such as a non-representative sample, sample size, overfitting, missing data) and their effects on any biases, statistical uncertainty, and generalizability	11
Usability of the model in the context of current care	27a	D	Describe how poor quality or unavailable input data (e.g., predictor values) should be assessed and handled when implementing the prediction model	11
	27b	D	Specify whether users will be required to interact in the handling of the input data or use of the model, and what level of expertise is required of users	NA
	27c	D;E	Discuss any next steps for future research, with a specific view to applicability and generalizability of the model	11

[4] This relates to the analysis code, for example, any data cleaning, feature engineering, model building, evaluation.
[5] This relates to the code to implement the model to get estimates of risk for a new individual.

From: Collins GS, Moons KGM, Dhiman P, et al. *BMJ* 2024;385:e078378. doi:10.1136/bmj-2023-078378

Supplemental Materials S2. List of features.

#	Features	Time point	RICD missing values, %*	Challenge-1 missing values, %*	Challenge-2 missing values, %*
1	age	baseline	0	0	0
2	sex	baseline	0	0	0
3	leucocytes	12h period	81.2	41.3	50.1
4	platelets	12h period	81.6	41.1	49.8
5	C-reactive protein	12h period	84.3	100	100
6	hemoglobin	12h period	94.0	37.3	47.7
7	albumin	12h period	87.4	100	100
8	lactate	12h period	96.2	100	100
9	pH	12h period	96.9	51.8	85.6
10	diabetis_2_type	baseline	0	100	100
11	chronic_kidney_disease	baseline	0	100	100
12	chronic obstructive pulmonary disease	baseline	0	100	100
13	coronary_artery_disease	baseline	0	100	100
14	arterial_hypertension	baseline	0	100	100
15	heart_failure	baseline	0	100	100
16	ischemic_stroke	baseline	0	100	100
17	hemorrhagic_stroke	baseline	0	100	100
18	traumatic_brain_injury	baseline	0	100	100
19	avg_heart_rate	1h period	29.4	7.8	12.1
20	min_heart_rate	1h period	29.4	7.8	12.1
21	max_heart_rate	1h period	29.4	7.8	12.1
22	sd_heart_rate	1h period	32.1	10.9	16.2
23	delta_3h_heart_rate	3h period	33.0	15.6	21.9
24	avg_respiratory_rate	1h period	43.6	9.8	21.1
25	min_respiratory_rate	1h period	43.6	9.8	21.1
26	max_respiratory_rate	1h period	43.6	9.8	21.1
27	sd_respiratory_rate	1h period	47.7	13.2	26.5
28	delta_3h_respiratory_rate	3h period	49.0	18.2	33.7
29	avg_temperature	1h period	40.3	66.2	66.1
30	min_temperature	1h period	40.3	66.2	66.1
31	max_temperature	1h period	40.3	66.2	66.1
32	sd_temperature	1h period	46.8	84.1	83.4
33	delta_3h_temperature	3h period	48.3	85.8	85.6
34	avg_systolic_BP	1h period	28.1	15.2	13.9
35	min_systolic_BP	1h period	28.1	15.2	13.9
36	max_systolic_BP	1h period	28.1	15.2	13.9
37	sd_systolic_BP	1h period	31.5	18.5	18.4
38	delta_3h_systolic_BP	3h period	33.3	23.6	24.3
39	avg_diastolic_BP	1h period	28.1	48.1	14.0
40	min_diastolic_BP	1h period	28.1	48.1	14.0
41	max_diastolic_BP	1h period	28.1	48.1	14.0
42	sd_diastolic_BP	1h period	31.5	50.1	18.4
43	delta_3h_diastolic_BP	3h period	33.3	52.6	24.3
44	avg_mean_BP	1h period	40.0	10.3	14.8
45	min_mean_BP	1h period	40.0	10.3	14.8
46	max_mean_BP	1h period	40.0	10.3	14.8
47	sd_mean_BP	1h period	40.8	13.8	19.0
48	delta_3h_mean_BP	3h period	42.2	19.2	24.9
49	avg_mean_SpO2	1h period	32.8	12.1	14.1
50	min_mean_SpO2	1h period	32.8	12.1	14.1
51	max_mean_SpO2	1h period	32.8	12.1	14.1
52	sd_mean_SpO2	1h period	37.2	15.9	18.6
53	delta_3h_mean_SpO2	3h period	38.6	21.4	24.4

*of all time windows

Table S1. Comparative characteristics of the three datasets.

Parameters	RICD	Challenge-1*	Challenge-2*	
Center	Federal Research and Clinical Center of Intensive Care Medicine and Rehabilitology, Russia	Beth Israel Deaconess Medical Center, USA	Emory University Hospital, USA	
Total number of patients incl.	575	20336	20000	
Sepsis during ICU stay	336	1790	1142	
No sepsis during ICU stay	239	18546	18858	
Patient-hours	388914	780886	754598	
Number of positive windows	2345	12458	7891	
Negative windows from positive cases	105224 (27.2% of negatives)	86738 (11.3% of negatives)	56585 (7.6% of negatives)	
Negative windows from negative cases	281345 (72.8% of negatives)	681690 (88.7% of negatives)	690122 (92.4% of negatives)	
Total number of negative windows	386569	768428	746707	
Sepsis prevalence (patients)	58.4%	8.8%	5.7%	
Sepsis prevalence (patient-hours)	0.6%	1.6%	1.0%	
				**p-value**
Time to sepsis onset, h.	N = 336, 233 (IQR 103; 402)	N = 1790, 35 (IQR 14; 78)	N = 1142, 35 (IQR 11; 81)	<0.001[1]
Sex, M (%)	N = 320, 55.7%	N = 11775, 57.9%	N = 10660, 53.3%	<0.001[2]
Age, years	N = 575, 61 (IQR 48; 73)	N = 20336, 65 (IQR 52; 76)	N = 20000, 62 (IQR 50; 72)	<0.001[1]
ICU stay, days	N = 575, 42 (IQR 30; 59)	N/D	N/D	-
Total hospital stay, days	N = 575, 57 (IQR 41; 71)	N/D	N/D	
**Laboratory parameters on admission**				
Hb, g/l	N = 436, 97 (IQR 87; 109)	N = 19632, 105 (IQR 95; 117)	N = 17900, 101 (IQR 86; 119)	<0.001[1]
Leukocytes, 10^9/L	N = 573, 9 (IQR 7; 13)	N = 19516, 10.9 (IQR 8.1; 14.3)	N = 17840, 9.6 (IQR 7.1; 12.8)	<0.001[1]
Platelets, 10^9/L	N = 573, 284 (IQR 213; 372)	N = 19557, 188 (IQR 136; 253)	N = 17850, 184 (IQR 131; 244)	<0.001[1]

Abbreviations: ICU, intensive care unit; IQR, interquartile range.

*The PhysioNet/Computing in Cardiology Challenge 2019 datasets.

1 - Kruskal-Wallis test; 2 - Chi-square test.

N – number of patients.

p-value <0.001 for all post-hoc analyses.

Table S2. Detailed characteristics of patients (RICD dataset).

Parameters	No sepsis during ICU stay N = 239	Sepsis during ICU stay N = 336	p-value
Time to sepsis onset, h.	-	233 (IQR 10; 402), 27 to 4044	-
Sex M	109, 45.6%	146, 43.5%	0.6[1]
F	130, 54.4%	190, 56.5%	
Age, years	59 (IQR 46; 69)	64 (IQR 48; 74)	0.007[2]
BMI, kg/m2	N = 227, 24.8 (IQR 21.6; 27.9)	N = 284, 24.8 (IQR 22.0; 28.8)	0.4[2]
Transfer from another hospital	237, 99.2%	330, 98.2%	0.5[3]
#Pneumonia on admission	136, 56.9%	243, 72.3%	**< 0.001**[1]
**Scale scores on admission***			
SOFA, score	N = 231, 4 (IQR 3; 5)	N = 330, 4 (IQR 3; 5)	0.8[2]
SIRS, score	N = 239, 1 (IQR 1; 2)	N = 336, 1 (IQR 1; 2)	0.5[2]
FOUR, score	N = 232, 13 (IQR 11; 15)	N = 311, 13 (IQR 10; 15)	0.5[2]
GCS, score	N = 230, 11 (IQR 9; 11)	N = 322, 11 (IQR 9; 13)	0.2[2]
CRS-R, score	N = 65, 10 (IQR 6; 16)	N = 207, 12 (IQR 6; 17)	0.3[2]
DRS, score	N = 223, 22 (IQR 19; 24)	N = 311, 21 (IQR 18; 24)	0.6[2]
**Laboratory parameters on admission***			
Hb, g/l	N = 163, 103 (IQR 95; 120)	N = 273, 106 (IQR 94; 120)	0.3[2]
Leukocytes, 10^9/L	N = 238, 9.5 (IQR 7.3; 12.2)	N = 335, 9.8 (IQR 7.4; 12.6)	0.4[2]
Neutrophils, 10^9/L	N = 238, 7.2 (IQR 5.2; 9.5)	N = 335, 7.5 (IQR 5.5; 10.3)	0.3[2]
Platelets, 10^9/L	N = 238, 299 (IQR 231; 383)	N = 335, 284 (IQR 215; 376)	0.1[2]
Lactate, mmol/l	N = 141, 1.1 (IQR 0.9; 1.6)	N = 243, 1.3 (IQR 0.9; 1.7)	0.028[2]
Creatinine, µmol/L	N = 236, 69.9 (IQR 55.4; 87.7)	N = 334, 75.7 (IQR 60.9; 99.7)	0.005[2]
CRP, mg/l	N = 209, 53.4 (IQR 29.0; 110.0)	N = 295, 56.7 (IQR 32.4; 107.9)	0.4[2]
Albumin, g/l	N = 212, 29.4 (IQR 25.8; 33.0)	N = 299, 29.6 (IQR 26.0; 33.0)	0.9[2]
Lymphocytes, 10^9/L	N = 238, 1.2 (IQR 0.9; 1.7)	N = 335, 1.2 (IQR 0.9; 1.6)	0.6[2]
Total protein, g/l	N = 237, 60.6 (IQR 55.0; 65.3)	N = 334, 59.7 (IQR 54.8; 64.8)	0.5[2]
Fibrinogen, g/l	N = 233, 5.2 (IQR 4.0; 6.8)	N = 332, 5.1 (IQR 4.1; 6.6)	0.8[2]
Procalcitonin, ng/ml	N = 15, 0.2 (IQR 0.1; 0.4)	N = 23, 0.3 (IQR 0.1; 0.6)	0.3[2]
D-dimer, mg/l	N = 7, 7.2 (IQR 2.8; 36.9)	N = 22, 2.9 (IQR 1.7; 4.5)	0.055[2]
pH of arterial blood	N = 140, 7.48 (IQR 7.45; 7.51)	N = 249, 7.47 (IQR 7.43; 7.51)	0.08[2]
**Comorbidity on admission**			
Ischaemic stroke	87, 36.4%	153, 45.5%	0.029[1]
Haemorrhagic stroke	55, 23.0%	66, 19.6%	0.3[1]
Traumatic brain injury	46, 19.2%	64, 19.0%	0.9[1]
Type 2 diabetes mellitus	33, 13.8%	53, 15.8%	0.5[1]
CKD	13, 5.4%	46, 13.7%	0.001[3]
COPD	4, 1.7%	12, 3.6%	0.2[3]
CAD	112, 46.9%	209, 62.2%	**< 0.001**[1]
Arterial hypertension	188, 78.7%	269, 80.1%	0.7[1]
Heart failure	19, 7.9%	65, 19.3%	**< 0.001**[3]
**Outcomes and complications**			
All-cause mortality	18, 7.5%	47, 14.0%	0.016[3]
Septic shock	-	124, 36.9%	-
Nosocomial pneumonia	204, 85.4%	311, 92.6%	0.008[3]
Duration of hospitalisation in ICU, days.	37 (IQR 29; 54)	44 (IQR 30; 62)	0.008[2]
Total duration of hospitalisation, days.	56 (IQR 41; 70)	58 (IQR 40; 71)	0.7[2]
Need for MV	222, 92.9%	328, 97.6%	0.011[3]
Use of vasopressors/inotropes	69, 28.9%	151, 44.9%	**< 0.001**[1]

Abbreviations: CRS-R, Coma Recovery Scale-Revised; DRS, Disability Rating Scale; FOUR, Full Outline of UnResponsiveness; IQR, interquartile range; SIRS, Systemic Inflammatory Response Syndrome; SOFA, Sequential Organ Failure Assessment; MV, mechanical ventilation; BMI, body mass index; ICU, intensive care unit; CRP, C-reactive protein; CKD, chronic kidney disease; CAD, coronary artery disease; COPD, chronic obstructive pulmonary disease; GCS, Glasgow Coma Scale.

1 - Chi-square test; 2 - Mann-Whitney U-test; 3 - Fisher's Exact test.

*If there were several assessments on admission (in the first 48 hours), the earliest one was included in the analysis.

$p < 0.001$ considered statistically significant (with Bonferroni correction).

#Pneumonia at admission, despite its significant association with sepsis, was not considered as a predictor due to variability in diagnostic criteria, which posed challenges to the clinical applicability of the model.

Table S3. Characteristics of vital and lab parameters (RICD dataset).

Parameters		Negative windows<br>N = 386569	Positive windows<br>N = 2345
HR	avg_heart_rate	N = 272465, 78 (IQR 69 to 89), 80 (SD 15)	N = 1923, 81 (IQR 72 to 91), 82 (SD 15)
	min_heart_rate	N = 272465, 75 (IQR 66 to 85), 76 (SD 15)	N = 1923, 77 (IQR 68 to 87), 78 (SD 14)
	max_heart_rate	N = 272465, 82 (IQR 73 to 93), 83 (SD 16)	N = 1923, 85 (IQR 74 to 96), 86 (SD 18)
	sd_heart_rate	N = 262065, 2.9 (IQR 1.6 to 5.2), 4.2 (SD 4.5)	N = 1838, 2.8 (IQR 1.5 to 5.1), 4.3 (SD 5.5)
	delta_3h_heart_rate	N = 258692, 0 (IQR -4.2 to 4.1), 0 (SD 10.1)	N = 1826, 0.1 (IQR -3.8 to 4.6), 0.5 (SD 11.7)
RR	avg_respiratory_rate	N = 218693, 17 (IQR 15 to 18), 17 (SD 4)	N = 759, 17 (IQR 17 to 18), 18 (SD 4)
	min_respiratory_rate	N = 218693, 16 (IQR 14 to 17), 16 (SD 3)	N = 759, 17 (IQR 16 to 18), 17 (SD 3)
	max_respiratory_rate	N = 218693, 17 (IQR 16 to 19), 18 (SD 4)	N = 759, 18 (IQR 17 to 19), 19 (SD 5)
	sd_respiratory_rate	N = 202697, 0.8 (IQR 0.4 to 1.6), 1.3 (SD 1.6)	N = 577, 0.6 (IQR 0.1 to 1.5), 1.2 (SD 1.6)
	delta_3h_respiratory_rate	N = 197764, 0 (IQR -1 to 1), 0 (SD 3.2)	N = 570, 0 (IQR -1 to 1), -0.1 (SD 3.2)
Temp.	avg_temperature	N = 231314, 37 (IQR 37 to 37), 37 (SD 1)	N = 1037, 37 (IQR 37 to 37), 37 (SD 1)
	min_temperature	N = 231314, 37 (IQR 37 to 37), 37 (SD 1)	N = 1037, 37 (IQR 37 to 37), 37 (SD 1)
	max_temperature	N = 231314, 37 (IQR 37 to 37), 37 (SD 1)	N = 1037, 37 (IQR 37 to 37), 37 (SD 1)
	sd_temperature	N = 206394, 0.1 (IQR 0 to 0.1), 0.1 (SD 0.3)	N = 697, 0.1 (IQR 0.1 to 0.2), 0.2 (SD 0.4)
	delta_3h_temperature	N = 200564, 0 (IQR -0.1 to 0.1), 0 (SD 0.5)	N = 679, 0 (IQR -0.1 to 0.1), 0 (SD 0.6)
SBP	avg_systolic_BP	N = 277999, 124 (IQR 111 to 138), 125 (SD 19)	N = 1806, 122 (IQR 108 to 135), 123 (SD 20)
	min_systolic_BP	N = 277999, 116 (IQR 103 to 130), 117 (SD 20)	N = 1806, 114 (IQR 100 to 126), 115 (SD 19)
	max_systolic_BP	N = 277999, 132 (IQR 118 to 147), 133 (SD 21)	N = 1806, 129 (IQR 114 to 145), 131 (SD 22)
	sd_systolic_BP	N = 264668, 7.4 (IQR 4.2 to 12), 9.1 (SD 7)	N = 1686, 7.3 (IQR 4.2 to 12.1), 9.4 (SD 7.8)
	delta_3h_systolic_BP	N = 257893, 0.5 (IQR -9 to 10), 0 (SD 17.6)	N = 1643, 1.5 (IQR -8.3 to 11), 0.8 (SD 18.5)
DBP	avg_diastolic_BP	N = 278004, 75 (IQR 67 to 84), 76 (SD 13)	N = 1805, 72 (IQR 64 to 81), 73 (SD 13)
	min_diastolic_BP	N = 278004, 70 (IQR 61 to 79), 70 (SD 14)	N = 1805, 67 (IQR 59 to 76), 68 (SD 14)
	max_diastolic_BP	N = 278004, 81 (IQR 72 to 91), 81 (SD 14)	N = 1805, 77 (IQR 69 to 88), 79 (SD 15)
	sd_diastolic_BP	N = 264673, 5.1 (IQR 3 to 8.4), 6.4 (SD 4.9)	N = 1684, 5 (IQR 2.6 to 8.5), 6.4 (SD 5.3)
	delta_3h_diastolic_BP	N = 257907, 0 (IQR -6 to 7), 0 (SD 12.2)	N = 1642, 1 (IQR -5 to 7), 0.8 (SD 12.2)
MBP	avg_mean_BP	N = 231896, 95 (IQR 85 to 106), 96 (SD 15)	N = 1516, 93 (IQR 83 to 105), 94 (SD 15)
	min_mean_BP	N = 231896, 88 (IQR 78 to 99), 89 (SD 16)	N = 1516, 85 (IQR 76 to 95), 87 (SD 15)
	max_mean_BP	N = 231896, 103 (IQR 91 to 115), 103 (SD 17)	N = 1516, 100 (IQR 89 to 114), 102 (SD 18)
	sd_mean_BP	N = 228636, 6.4 (IQR 3.6 to 10.4), 7.9 (SD 6)	N = 1473, 6.4 (IQR 3.8 to 11.1), 8.4 (SD 6.7)
	delta_3h_mean_BP	N = 223175, 0 (IQR -8 to 8), 0 (SD 15)	N = 1443, 1 (IQR -7 to 9), 0.9 (SD 15.9)
SpO2	avg_mean_SpO2	N = 259867, 99 (IQR 97 to 99), 97 (SD 3)	N = 1481, 99 (IQR 97 to 100), 98 (SD 3)
	min_mean_SpO2	N = 259867, 98 (IQR 96 to 99), 96 (SD 5)	N = 1481, 98 (IQR 96 to 99), 97 (SD 5)
	max_mean_SpO2	N = 259867, 99 (IQR 98 to 100), 98 (SD 3)	N = 1481, 99 (IQR 98 to 100), 99 (SD 2)
	sd_mean_SpO2	N = 243110, 0.5 (IQR 0 to 1.1), 1.1 (SD 2.1)	N = 1308, 0.5 (IQR 0.1 to 1), 1.1 (SD 2.2)
	delta_3h_mean_SpO2	N = 237416, 0 (IQR -0.5 to 0.5), 0 (SD 3.8)	N = 1290, 0 (IQR -0.8 to 0.5), -0.1 (SD 3.7)
Leukocytes, 10^9/L		N = 73206, 9.3 (IQR 7 to 13), 42.9 (SD 231.6)	N = 361, 10.4 (IQR 7.4 to 15.1), 85.9 (SD 307.9)
Platelets, 10^9/L		N = 71647, 284 (IQR 213 to 372), 297.2 (SD 122.3)	N = 354, 226 (IQR 139 to 322), 235.8 (SD 116.2)
CRP, mg/l		N = 60868, 47.6 (IQR 25.7 to 99.3), 70.2 (SD 65.2)	N = 311, 75 (IQR 35 to 139.8), 94.1 (SD 79.1)
Hb, g/l		N = 23238, 97 (IQR 87 to 109), 99 (SD 18.6)	N = 57, 111 (IQR 92 to 116), 105.4 (SD 16.9)
Albumin, g/l		N = 49144, 27.7 (IQR 24.2 to 31.1), 27.7 (SD 5)	N = 226, 28 (IQR 24 to 30.7), 27.6 (SD 4.3)
Lactate, mmol/l		N = 14701, 1.2 (IQR 0.9 to 1.7), 1.5 (SD 1.2)	N = 107, 1.3 (IQR 1.1 to 1.8), 2.8 (SD 4)
pH of arterial blood		N = 12182, 7.5 (IQR 7.5 to 7.5), 7.5 (SD 0.1)	N = 105, 7.5 (IQR 7.4 to 7.5), 7.5 (SD 0.1)

Abbreviations: avg, average over three hours (data collection window); min, min over three hours; max, maximum over three hours; sd, standard deviation over three hours; delta_3h, average over the last hour X in the observation window minus the average over X-2 hours; IQR, interquartile range; BP, blood pressure; HR, heart rate; RR, respiratory rate; SBP, systolic blood pressure; DBP, diastolic blood pressure; MBP, mean blood pressure.
Positive cases: N = 336, negative cases: N = 575.
Negative windows - no sepsis in the 6 h prediction period, positive windows - assessments in the period ≤6 h before sepsis developed.

Table S4. Characteristics of vital and lab parameters (Challenge-1 dataset).

Parameters		Negative windows N = 780886	Positive windows N = 12458
HR	avg_heart_rate	N = 708663, 84 (IQR 73 to 95), 85 (SD 16)	N = 11535, 89 (IQR 77 to 102), 90 (SD 18)
	min_heart_rate	N = 708663, 80 (IQR 70 to 91), 81 (SD 16)	N = 11535, 85 (IQR 73 to 98), 86 (SD 18)
	max_heart_rate	N = 708663, 88 (IQR 77 to 100), 89 (SD 17)	N = 11535, 93 (IQR 81 to 107), 94 (SD 19)
	sd_heart_rate	N = 684346, 3.2 (IQR 1.6 to 5.5), 4.2 (SD 3.9)	N = 11202, 3.5 (IQR 1.8 to 5.9), 4.6 (SD 4.4)
	delta_3h_heart_rate	N = 648234, 0 (IQR -4 to 4), -0.1 (SD 9)	N = 10640, 0 (IQR -4 to 5), 0.4 (SD 10.1)
RR	avg_respiratory_rate	N = 692968, 18 (IQR 15 to 21), 19 (SD 5)	N = 11362, 20 (IQR 17 to 24), 21 (SD 6)
	min_respiratory_rate	N = 692968, 16 (IQR 14 to 19), 17 (SD 5)	N = 11362, 18 (IQR 15 to 22), 19 (SD 5)
	max_respiratory_rate	N = 692968, 20 (IQR 17 to 24), 21 (SD 6)	N = 11362, 22 (IQR 18 to 27), 23 (SD 6)
	sd_respiratory_rate	N = 666609, 2 (IQR 1 to 3.3), 2.4 (SD 2)	N = 10995, 1.9 (IQR 1 to 3.2), 2.4 (SD 2.2)
	delta_3h_respiratory_rate	N = 627994, 0 (IQR -2 to 2.5), 0.1 (SD 4.7)	N = 10410, 0 (IQR -2 to 2), 0.1 (SD 4.8)
Temp.	avg_temperature	N = 259944, 37 (IQR 37 to 38), 37 (SD 1)	N = 3972, 37 (IQR 37 to 38), 37 (SD 1)
	min_temperature	N = 259944, 37 (IQR 36 to 37), 37 (SD 1)	N = 3972, 37 (IQR 37 to 38), 37 (SD 1)
	max_temperature	N = 259944, 37 (IQR 37 to 38), 37 (SD 1)	N = 3972, 37 (IQR 37 to 38), 37 (SD 1)
	sd_temperature	N = 122522, 0.1 (IQR 0.1 to 0.2), 0.2 (SD 0.2)	N = 1597, 0.2 (IQR 0.1 to 0.4), 0.3 (SD 0.3)
	delta_3h_temperature	N = 109324, 0.1 (IQR -0.1 to 0.3), 0.1 (SD 0.5)	N = 1318, 0.1 (IQR -0.2 to 0.3), 0.1 (SD 0.6)
SBP	avg_systolic_BP	N = 652112, 119 (IQR 107 to 133), 121 (SD 20)	N = 10130, 119 (IQR 105 to 135), 121 (SD 21)
	min_systolic_BP	N = 652112, 111 (IQR 99 to 126), 113 (SD 20)	N = 10130, 110 (IQR 97 to 126), 113 (SD 21)
	max_systolic_BP	N = 652112, 126 (IQR 113 to 142), 129 (SD 22)	N = 10130, 128 (IQR 112 to 144), 130 (SD 23)
	sd_systolic_BP	N = 626546, 7.1 (IQR 4 to 11.4), 8.6 (SD 6.4)	N = 9808, 7.5 (IQR 4.2 to 12.3), 9.3 (SD 7.2)
	delta_3h_systolic_BP	N = 587298, 0 (IQR -9 to 9), 0 (SD 16.1)	N = 9230, 0 (IQR -9.5 to 9.5), -0.3 (SD 18)
DBP	avg_diastolic_BP	N = 397794, 59 (IQR 52 to 67), 60 (SD 11)	N = 7105, 60 (IQR 53 to 68), 61 (SD 12)
	min_diastolic_BP	N = 397794, 55 (IQR 48 to 62), 56 (SD 11)	N = 7105, 55 (IQR 48 to 63), 56 (SD 12)
	max_diastolic_BP	N = 397794, 63 (IQR 55 to 72), 64 (SD 13)	N = 7105, 64 (IQR 56 to 74), 66 (SD 14)
	sd_diastolic_BP	N = 383059, 3.6 (IQR 2.1 to 6), 4.7 (SD 4.2)	N = 6866, 4 (IQR 2.1 to 6.7), 5.2 (SD 4.6)
	delta_3h_diastolic_BP	N = 363393, 0 (IQR -5 to 5), -0.1 (SD 9.5)	N = 6509, 0 (IQR -5 to 5), -0.1 (SD 10.5)
MBP	avg_mean_BP	N = 689438, 77 (IQR 69 to 87), 79 (SD 13)	N = 11330, 77 (IQR 69 to 87), 79 (SD 14)
	min_mean_BP	N = 689438, 72 (IQR 64 to 81), 73 (SD 13)	N = 11330, 72 (IQR 64 to 81), 73 (SD 14)
	max_mean_BP	N = 689438, 83 (IQR 74 to 93), 85 (SD 16)	N = 11330, 83 (IQR 74 to 95), 85 (SD 17)
	sd_mean_BP	N = 662350, 4.9 (IQR 2.8 to 8.1), 6.2 (SD 5.7)	N = 10971, 5.3 (IQR 3 to 8.8), 6.9 (SD 6.5)
	delta_3h_mean_BP	N = 620407, 0 (IQR -6 to 6), -0.1 (SD 12.6)	N = 10318, 0 (IQR -7 to 7), -0.1 (SD 14.1)
SpO2	avg_mean_SpO2	N = 675514, 98 (IQR 96 to 99), 97 (SD 3)	N = 11159, 98 (IQR 96 to 99), 97 (SD 3)
	min_mean_SpO2	N = 675514, 97 (IQR 95 to 99), 96 (SD 3)	N = 11159, 97 (IQR 94 to 99), 96 (SD 3)
	max_mean_SpO2	N = 675514, 99 (IQR 97 to 100), 98 (SD 2)	N = 11159, 99 (IQR 97 to 100), 98 (SD 2)
	sd_mean_SpO2	N = 645601, 0.7 (IQR 0.5 to 1.5), 1.1 (SD 1.4)	N = 10764, 0.8 (IQR 0.6 to 1.5), 1.2 (SD 1.5)
	delta_3h_mean_SpO2	N = 603675, 0 (IQR -1 to 1), -0.1 (SD 2.6)	N = 10117, 0 (IQR -1 to 1), -0.1 (SD 2.8)
Leukocytes, 10^9/L		N = 458641, 10.9 (IQR 8.1 to 14.3), 11.9 (SD 7)	N = 6490, 11.7 (IQR 8.6 to 15.7), 13.1 (SD 8.2)
Platelets, 10^9/L		N = 460090, 188 (IQR 136 to 253), 206.7 (SD 108.6)	N = 6460, 191 (IQR 127 to 270), 214.8 (SD 129.1)
Hb, g/l		N = 489242, 105 (IQR 95 to 117), 106.7 (SD 17.3)	N = 6846, 102 (IQR 92 to 115), 104.1 (SD 17.8)
pH of arterial blood		N = 376709, 7.4 (IQR 7.4 to 7.4), 7.4 (SD 0.1)	N = 7004, 7.4 (IQR 7.4 to 7.4), 7.4 (SD 0.1)

Abbreviations: avg, average over three hours (data collection window); min, min over three hours; max, maximum over three hours; sd, standard deviation over three hours; delta_3h, average over the last hour X in the observation window minus the average over X-2 hours; IQR, interquartile range; BP, blood pressure; HR, heart rate; RR, respiratory rate; SBP, systolic blood pressure; DBP, diastolic blood pressure; MBP, mean blood pressure.

Positive cases: N = 1790, negative cases: N = 20336.

Negative windows - no sepsis in the 6 h prediction period, positive windows - assessments in the period ≤6 h before sepsis developed.

Table S5. Characteristics of vital and lab parameters (Challenge-2 dataset).

Parameters		Negative windows N = 754598	Positive windows N = 7891
HR	avg_heart_rate	N = 656138, 83 (IQR 72 to 95), 84 (SD 17)	N = 6985, 91 (IQR 78 to 103), 91 (SD 19)
	min_heart_rate	N = 656138, 80 (IQR 68 to 91), 81 (SD 17)	N = 6985, 86 (IQR 74 to 100), 87 (SD 19)
	max_heart_rate	N = 656138, 87 (IQR 75 to 99), 88 (SD 18)	N = 6985, 95 (IQR 81 to 108), 96 (SD 20)
	sd_heart_rate	N = 625379, 3.1 (IQR 1.5 to 5.3), 4.1 (SD 4)	N = 6667, 3.1 (IQR 1.5 to 6.1), 4.7 (SD 4.9)
	delta_3h_heart_rate	N = 583323, 0 (IQR -4 to 4), -0.1 (SD 8.9)	N = 6167, 0 (IQR -4 to 4), 0.2 (SD 10.9)
RR	avg_respiratory_rate	N = 589214, 18 (IQR 16 to 21), 19 (SD 4)	N = 5901, 20 (IQR 16 to 23), 20 (SD 6)
	min_respiratory_rate	N = 589214, 17 (IQR 14 to 19), 17 (SD 4)	N = 5901, 18 (IQR 15 to 21), 18 (SD 5)
	max_respiratory_rate	N = 589214, 20 (IQR 18 to 23), 20 (SD 5)	N = 5901, 21 (IQR 18 to 25), 22 (SD 6)
	sd_respiratory_rate	N = 548884, 1.5 (IQR 1 to 2.6), 2 (SD 1.9)	N = 5451, 1.5 (IQR 0.7 to 2.8), 2.1 (SD 2.1)
	delta_3h_respiratory_rate	N = 495293, 0 (IQR -2 to 2), 0 (SD 4.1)	N = 4841, 0 (IQR -2 to 2), 0.1 (SD 4.5)
Temp.	avg_temperature	N = 252821, 37 (IQR 36 to 37), 37 (SD 1)	N = 2867, 37 (IQR 37 to 38), 37 (SD 1)
	min_temperature	N = 252821, 37 (IQR 36 to 37), 37 (SD 1)	N = 2867, 37 (IQR 37 to 38), 37 (SD 1)
	max_temperature	N = 252821, 37 (IQR 37 to 38), 37 (SD 1)	N = 2867, 37 (IQR 37 to 38), 37 (SD 1)
	sd_temperature	N = 123680, 0.1 (IQR 0.1 to 0.2), 0.2 (SD 0.2)	N = 1622, 0.1 (IQR 0.1 to 0.2), 0.2 (SD 0.2)
	delta_3h_temperature	N = 106990, 0 (IQR -0.1 to 0.2), 0.1 (SD 0.4)	N = 1393, 0 (IQR -0.2 to 0.3), 0.1 (SD 0.5)
SBP	avg_systolic_BP	N = 642544, 125 (IQR 110 to 142), 127 (SD 23)	N = 6898, 122 (IQR 106 to 142), 125 (SD 25)
	min_systolic_BP	N = 642544, 116 (IQR 102 to 133), 119 (SD 23)	N = 6898, 112 (IQR 98 to 132), 115 (SD 25)
	max_systolic_BP	N = 642544, 133 (IQR 117 to 151), 135 (SD 25)	N = 6898, 132 (IQR 112 to 154), 134 (SD 29)
	sd_systolic_BP	N = 609491, 7.6 (IQR 4.3 to 12.3), 9.3 (SD 7.2)	N = 6563, 8.3 (IQR 4.6 to 14.1), 10.8 (SD 9.2)
	delta_3h_systolic_BP	N = 565088, 0 (IQR -10 to 10), -0.2 (SD 17.9)	N = 6054, 0 (IQR -11 to 10), -1 (SD 21.5)
DBP	avg_diastolic_BP	N = 642358, 65 (IQR 57 to 74), 66 (SD 13)	N = 6893, 63 (IQR 55 to 73), 64 (SD 13)
	min_diastolic_BP	N = 642358, 60 (IQR 53 to 69), 62 (SD 13)	N = 6893, 58 (IQR 51 to 67), 59 (SD 13)
	max_diastolic_BP	N = 642358, 70 (IQR 61 to 80), 71 (SD 15)	N = 6893, 67 (IQR 59 to 79), 70 (SD 16)
	sd_diastolic_BP	N = 609294, 4.3 (IQR 2.5 to 7.2), 5.6 (SD 5.3)	N = 6558, 4.2 (IQR 2.3 to 7.6), 5.9 (SD 6)
	delta_3h_diastolic_BP	N = 564809, 0 (IQR -5.5 to 5), -0.1 (SD 11.4)	N = 6046, 0 (IQR -6 to 5), -0.6 (SD 12.4)
MBP	avg_mean_BP	N = 636268, 85 (IQR 75 to 96), 86 (SD 15)	N = 6868, 82 (IQR 73 to 94), 84 (SD 16)
	min_mean_BP	N = 636268, 79 (IQR 70 to 90), 81 (SD 15)	N = 6868, 76 (IQR 67 to 88), 78 (SD 15)
	max_mean_BP	N = 636268, 90 (IQR 80 to 102), 92 (SD 18)	N = 6868, 88 (IQR 77 to 102), 91 (SD 19)
	sd_mean_BP	N = 605006, 5.1 (IQR 3 to 8.5), 6.5 (SD 5.8)	N = 6536, 5.5 (IQR 3.1 to 9.3), 7.2 (SD 7.2)
	delta_3h_mean_BP	N = 560686, 0 (IQR -6.5 to 6.5), -0.1 (SD 13)	N = 6026, -1 (IQR -8 to 6), -0.8 (SD 15.2)
SpO2	avg_mean_SpO2	N = 640978, 98 (IQR 96 to 99), 97 (SD 3)	N = 6868, 97 (IQR 95 to 99), 97 (SD 3)
	min_mean_SpO2	N = 640978, 97 (IQR 94 to 99), 96 (SD 4)	N = 6868, 96 (IQR 94 to 99), 96 (SD 4)
	max_mean_SpO2	N = 640978, 99 (IQR 97 to 100), 98 (SD 2)	N = 6868, 99 (IQR 97 to 100), 98 (SD 3)
	sd_mean_SpO2	N = 607488, 0.8 (IQR 0.5 to 1.5), 1.2 (SD 1.5)	N = 6528, 1 (IQR 0.5 to 1.7), 1.3 (SD 1.8)
	delta_3h_mean_SpO2	N = 564387, 0 (IQR -1 to 1), -0.1 (SD 2.8)	N = 6008, 0 (IQR -1 to 1), 0 (SD 3.3)
Leukocytes, 10^9/L		N = 376532, 9.6 (IQR 7.1 to 12.8), 10.6 (SD 7.9)	N = 4572, 11.6 (IQR 8 to 16.1), 13.1 (SD 9.4)
Platelets, 10^9/L		N = 379105, 184 (IQR 131 to 244), 195.4 (SD 95.4)	N = 4613, 167 (IQR 107 to 247), 183.7 (SD 106.6)
Hb, g/l		N = 394339, 101 (IQR 86 to 119), 103.7 (SD 22.3)	N = 4707, 97 (IQR 84 to 112), 99.9 (SD 21.1)
pH of arterial blood		N = 108507, 7.4 (IQR 7.3 to 7.4), 7.4 (SD 0.1)	N = 2742, 7.4 (IQR 7.3 to 7.5), 7.4 (SD 0.1)

Abbreviations: avg, average over three hours (data collection window); min, min over three hours; max, maximum over three hours; sd, standard deviation over three hours; delta_3h, average over the last hour X in the observation window minus the average over X-2 hours; IQR, interquartile range; BP, blood pressure; HR, heart rate; RR, respiratory rate; SBP, systolic blood pressure; DBP, diastolic blood pressure; MBP, mean blood pressure.
Positive cases: N = 1142, negative cases: N = 20000.
Negative windows - no sepsis in the 6 h prediction period, positive windows - assessments in the period ≤6 h before sepsis developed.

Table S6. Comparative characteristics of patients with hypo- and hyperinflammatory sepsis phenotypes (RICD dataset).

Parameters	Hyperinflammatory sepsis N = 243	Hypoinflammatory sepsis N = 93	p-value
Time to sepsis onset, h.	206 (IQR 88; 359), 27 to 2225	334 (IQR 162; 521), 424 to 4044	**< 0.001[2]**
Sex, M	144, 59.3%	46, 49.5%	0.11[1]
Sex, F	99, 40.7%	47, 50.5%	
Age, years	64 (IQR 48; 74)	65 (IQR 48; 76)	0.6[2]
BMI, kg/m2	N = 208, 24.8 (IQR 21.7; 28.5)	N = 76, 25.1 (IQR 22.7; 30.8)	0.2[2]
Transfer from another hospital	238, 97.9%	92, 98.9%	0.9[3]
Pneumonia on admission	179, 73.7%	64, 68.8%	0.4[1]
**Scale scores on admission***			
SOFA, score	N = 239, 4 (IQR 3; 5)	N = 91, 3 (IQR 3; 5)	0.4[2]
SIRS, score	N = 243, 1 (IQR 1; 2)	N = 93, 1 (IQR 0; 2)	**0.001[2]**
FOUR, score	N = 224, 12 (IQR 10; 14)	N = 87, 13 (IQR 12; 16)	**< 0.001[2]**
GCS, score	N = 232, 10 (IQR 8; 12)	N = 90, 11 (IQR 10; 14)	**0.005[2]**
CRS-R, score	N = 155, 11 (IQR 6; 17)	N = 52, 14.5 (IQR 8; 18)	0.056[2]
DRS, score	N = 223, 22 (IQR 18; 24)	N = 88, 21 (IQR 18; 22)	**0.042[2]**
**Laboratory parameters on admission***			
Hb, g/l	N = 196, 105 (IQR 94; 120)	N = 77, 110 (IQR 97; 122)	0.3[2]
Leukocytes, 10^9/L	N = 242, 10.21 (IQR 7.73; 13.17)	N = 93, 8.90 (IQR 6.63; 11.16)	**< 0.001[2]**
Neutrophils, 10^9/L	N = 242, 7.9 (IQR 5.8; 10.6)	N = 93, 6.57 (IQR 4.54; 9.13)	**< 0.001[2]**
Platelets, 10^9/L	N = 242, 288 (IQR 215; 381)	N = 93, 279 (IQR 218; 354)	0.4[2]
Lactate, mmol/l	N = 177, 1.3 (IQR 0.9; 1.7)	N = 66, 1.25 (IQR 0.9; 1.7)	0.6[2]
Creatinine, µmol/L	N = 241, 76 (IQR 60.4; 101.5)	N = 93, 75.4 (IQR 62.3; 95.7)	0.8[2]
CRP, mg/l	N = 211, 63.09 (IQR 34.96; 121.76)	N = 84, 43.18 (IQR 27.15; 74.21)	**0.006[2]**
Albumin, g/l	N = 215, 29.6 (IQR 25.8; 32.8)	N = 84, 29.65 (IQR 26.9; 33.1)	0.4[2]
Lymphocytes, 10^9/L	N = 242, 1.2 (IQR 0.9; 1.62)	N = 93, 1.27 (IQR 0.9; 1.67)	0.6[2]
Total protein, g/l	N = 241, 59.6 (IQR 55.2; 64.3)	N = 93, 59.9 (IQR 54.7; 65.6)	0.5[2]
Fibrinogen, g/l	N = 241, 5.2 (IQR 4.2; 6.8)	N = 91, 5.0 (IQR 3.5; 6.2)	**0.034[2]**
Procalcitonin, ng/ml	N = 18, 0.35 (IQR 0.2; 0.7)	N = 5, 0.1 (IQR 0.1; 0.2)	**0.046[2]**
D-dimer, mg/l	N = 13, 2.9 (IQR 1.8; 3.1)	N = 9, 4.5 (IQR 1.7; 6.2)	0.4[2]
pH of arterial blood	N = 179, 7.47 (IQR 7.44; 7.51)	N = 70, 7.46 (IQR 7.43; 7.5)	0.3[2]
**Comorbidity on admission**			
Ischaemic stroke	110, 45.3%	43, 46.2%	0.9[1]
Haemorrhagic stroke	45, 18.5%	21, 22.6%	0.4[1]
Traumatic brain injury	49, 20.2%	15, 16.1%	0.4[1]
Type 2 diabetes mellitus	40, 16.5%	13, 14.0%	0.6[1]
CKD	36, 14.8%	10, 10.8%	0.3[1]
COPD	9, 3.7%	3, 3.2%	0.9[3]
CAD	150, 61.7%	59, 63.4%	0.8[1]
Arterial hypertension	195, 80.2%	74, 79.6%	0.9[1]
Heart failure	47, 19.3%	18, 19.4%	0.9[1]
**Outcomes and complications**			
All-cause mortality	39, 16.0%	8, 8.6%	0.082[3]
Septic shock	108, 44.4%	16, 17.2%	**< 0.001[1]**
Nosocomial pneumonia	232, 95.5%	79, 84.9%	**0.002[3]**
Duration of hospitalisation in ICU, days.	37 (IQR 29; 54)	44 (IQR 30; 62)	**0.008[2]**
Total duration of hospitalisation, days.	56 (IQR 41; 70)	58 (IQR 40; 71)	0.7[2]
Need for MV	239, 97.9%	90, 96.8%	0.7[3]
Use of vasopressors/inotropes	128, 52.7%	23, 24.7%	**< 0.001[1]**

Abbreviations: CRS-R, Coma Recovery Scale-Revised; DRS, Disability Rating Scale; FOUR, Full Outline of UnResponsiveness; IQR, interquartile range; SIRS, Systemic Inflammatory Response Syndrome; SOFA, Sequential Organ Failure Assessment; MV, mechanical ventilation; BMI, body mass index; ICU, intensive care unit; CRP, C-reactive protein; CKD, chronic kidney disease; CAD, coronary artery disease; COPD, chronic obstructive pulmonary disease; GCS, Glasgow Coma Scale.

1 - Chi-square test; 2 - Mann-Whitney U-test; 3 - Fisher's Exact test.

*If there were several assessments on admission (in the first 48 hours), the earliest one was included in the analysis.

Table S7. Comparative baseline characteristics of the train set, validation set, internal and external test sets (PCI/CCI sepsis prediction model).

Parameters	Train set	Validation set	Internal test set	External test set
Dataset	[RICD] (60% of data)	[RICD] (20% of data)	[RICD] (20% of data)	[Challenge-2*]
Center	Federal Research and Clinical Center of Intensive Care Medicine and Rehabilitology, Russia	Federal Research and Clinical Center of Intensive Care Medicine and Rehabilitology, Russia	Federal Research and Clinical Center of Intensive Care Medicine and Rehabilitology, Russia	Emory University Hospital, USA
Total number of patients	345	115	115	20000
Number of septic patients	195	68	73	1142
Patient-hours	238396	81621	68897	754598
Sepsis prevalence (patients)	56.5%	59.1%	63.5%	5.7%
Sepsis prevalence (patient-hours)	0.6%	0.6%	0.7%	1.0%
Time to sepsis onset, h.	N = 195, 206 (IQR 88; 402)	N = 68, 264 (IQR 123; 513)	N = 73, 402 (IQR 211; 886)	N = 1142, 29 (IQR 5; 75)
Sex, M (%)	N = 201, 58.3%	N = 61, 53.0%	N = 58, 50.4%	N = 10660, 53.3%
Age, years	N = 345, 60 (IQR 46; 72)	N = 115, 62 (IQR 51; 75)	N = 115, 64 (IQR 46; 71)	N = 20000, 62 (IQR 50; 72)

Abbreviations: IQR, interquartile range.
*The PhysioNet/Computing in Cardiology Challenge 2019 datasets.

Table S8. Comparative baseline characteristics of the train set, validation set, internal and external test sets (Universal sepsis prediction model).

Parameters	Train set	Validation set	Internal test set	External test set
Dataset	[RICD (80% of data) + Challenge-1] (80% of data)	[RICD (80% of data) + Challenge-1] (20% of data)	[RICD] (20% of data)	[Challenge-2*]
Center	Federal Research and Clinical Center of Intensive Care Medicine and Rehabilitology, Russia	Federal Research and Clinical Center of Intensive Care Medicine and Rehabilitology, Russia	Federal Research and Clinical Center of Intensive Care Medicine and Rehabilitology, Russia	Emory University Hospital, USA
Total number of patients	16552	4138	115	20000
Number of septic patients	1660	383	73	1142
Patient-hours	887165	213738	68897	754598
Sepsis prevalence (patients)	10.0%	9.3%	63.5%	5.7%
Sepsis prevalence (patient-hours)	1.3%	1.2%	0.7%	1.0%
Time to sepsis onset, h.	N = 1660, 40 (IQR 26; 49)	N = 383, 40 (IQR 26; 49)	N = 73, 402 (IQR 211; 886)	N = 1142, 29 (IQR 5; 75)
Sex, M (%)	N = 9666, 58.4%	N = 2337, 57.2%	N = 58, 50.4%	N = 10660, 53.3%
Age, years	N = 16552, 64 (IQR 52; 76)	N = 4138, 65 (IQR 52; 76)	N = 115, 64 (IQR 46; 71)	N = 20000, 62 (IQR 50; 72)

Abbreviations: IQR, interquartile range.
*The PhysioNet/Computing in Cardiology Challenge 2019 datasets.

Table S9. AUROC values (and 95% CIs) of machine learning models for 6-hour sepsis prediction across training, validation, and test sets.

Parameters	N (patients)	No of features	XGBoost	AdaBoost	LightGBM	RandomForest
**Prolonged/chronic critical illness sepsis prediction model**						
Train set	345	26	0.879 (0.873; 0.886)	0.813 (0.802; 0.823)	0.905 (0.899; 0.910)	0.815 (0.800; 0.821)
Validation set	115		0.711 (0.684; 0.729)	0.708 (0.686; 0.729)	0.693 (0.670; 0.716)	0.719 (0.700; 0.740)
Internal test set	115		0.752* (0.730; 0.767)	0.731 (0.710; 0.752)	0.731 (0.712; 0.751)	0.751 (0.728; 0.771)
External test set	20000		0.474 (0.467; 0.481)	0.471 (0.464; 0.478)	0.485 (0.479; 0.492)	0.511 (0.504; 0.517)
RICD dataset	575		0.819 (0.812; 0.826)	0.776 (0.767; 0.785)	0.831 (0.824; 0.838)	0.784 (0.775; 0.792)
**Universal sepsis prediction model**						
Train set	16552	25	0.765 (0.761; 0.769)	0.731 (0.726; 0.735)	0.756 (0.752; 0.760)	0.777 (0.773; 0.781)
Validation set	4138		0.700 (0.690; 0.710)	0.699 (0.689; 0.709)	0.698 (0.689; 0.708)	0.695 (0.686; 0.705)
Internal test set	115		0.752 (0.734; 0.771)	0.741 (0.720; 762)	0.754* (0.736; 0.773)	0.748 (0.729; 0.767)
External test set	20000		0.646 (0.639; 0.652)	0.634 (0.627; 0.641)	0.655 (0.648; 0.662)	0.632 (0.625; 0.638)
RICD dataset	575		0.796 (0.788; 0.804)	0.741 (0.731; 0.751)	0.802 (0.795; 0.810)	0.808 (0.800; 0.817)

*Model with maximum AUROC value on internal test set (no statistically significant differences).

Table S10. Performance characteristics of best machine learning model for 6-hour sepsis prediction (RICD dataset).

Model	AUROC (RICD)	95% CI	p-value	Cutoff for sepsis score	Sens.	Spec.	PPV#	NPV#	Acc.#	F1 score
XGBoost (PCI/CCI sepsis prediction model)	0.819	0.812; 0.826	**<0.001**	≥0.4725 (Youden's index)	86.5 (84.5; 88.2)	75.0 (74.9; 75.2)	16.2 (15.9; 16.5)	99.0 (98.9; 99.1)	75.6 (75.5; 75.8)	0.273
				≥0.5651 (max LR+ & Sens. ≥70%)	70.9 (68.4; 73.3)	83.1 (82.9; 83.2)	19.0 (18.5; 19.5)	98.1 (97.9; 98.2)	82.4 (82.3; 82.6)	0.300
				≥0.6331 (max LR+ & Sens. ≥60%)	60.0 (57.4; 62.6)	87.4 (87.2; 87.5)	21.0 (20.3; 21.7)	97.5 (97.3; 97.7)	85.9 (85.8; 86.0)	0.311

Abbreviations: AUROC, area under the ROC curve; PCI, prolonged critical illness; CCI, chronic critical illness; CI, confidence interval; PPV, positive predictive value; NPV, negative predictive value; Acc, predictive accuracy.

#Prevalence-adjusted calculation (5.3% prevalence: 13316/252836-time windows).

Figure S1. Distribution of time to sepsis onset after ICU admission in three datasets.

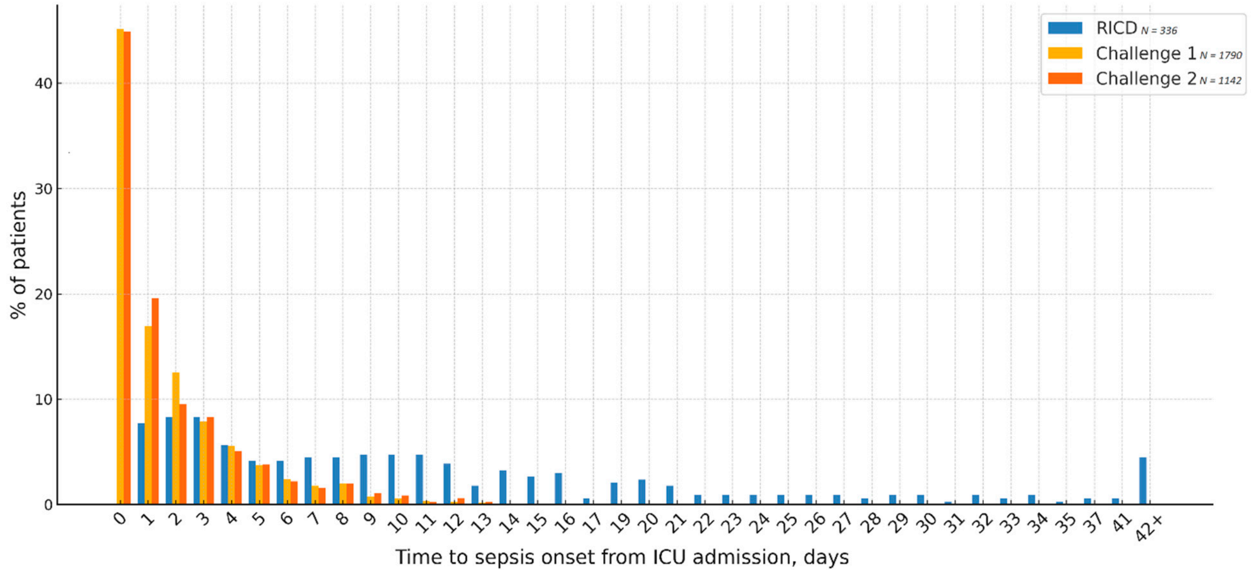

Figure S2. ROC curves of the best-performing machine learning models for early sepsis prediction (6-hour window, external validation).

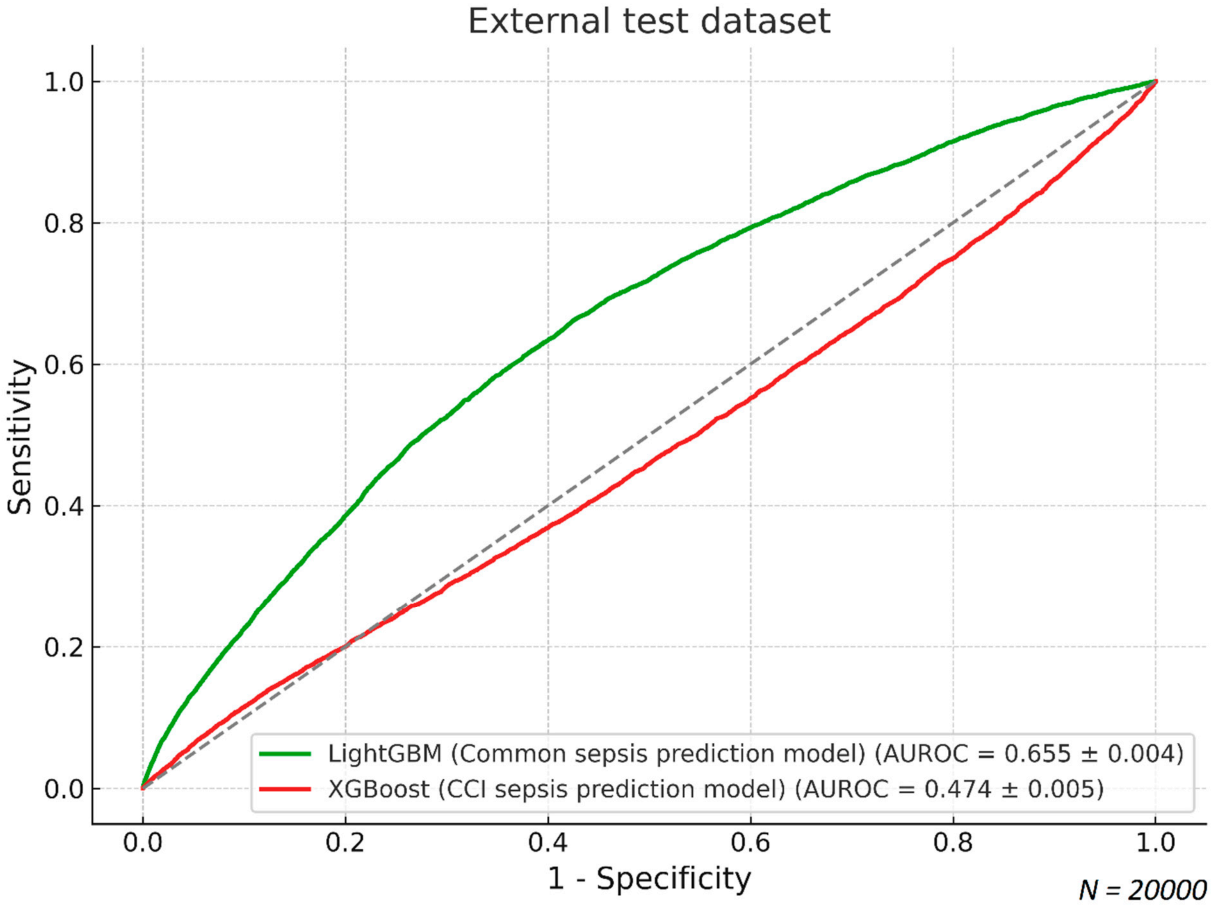

Abbreviations: CCI, chronic critical illness.

Figure S3. ROC curves of the best-performing machine learning models for early sepsis prediction (6-hour window, RICD dataset).

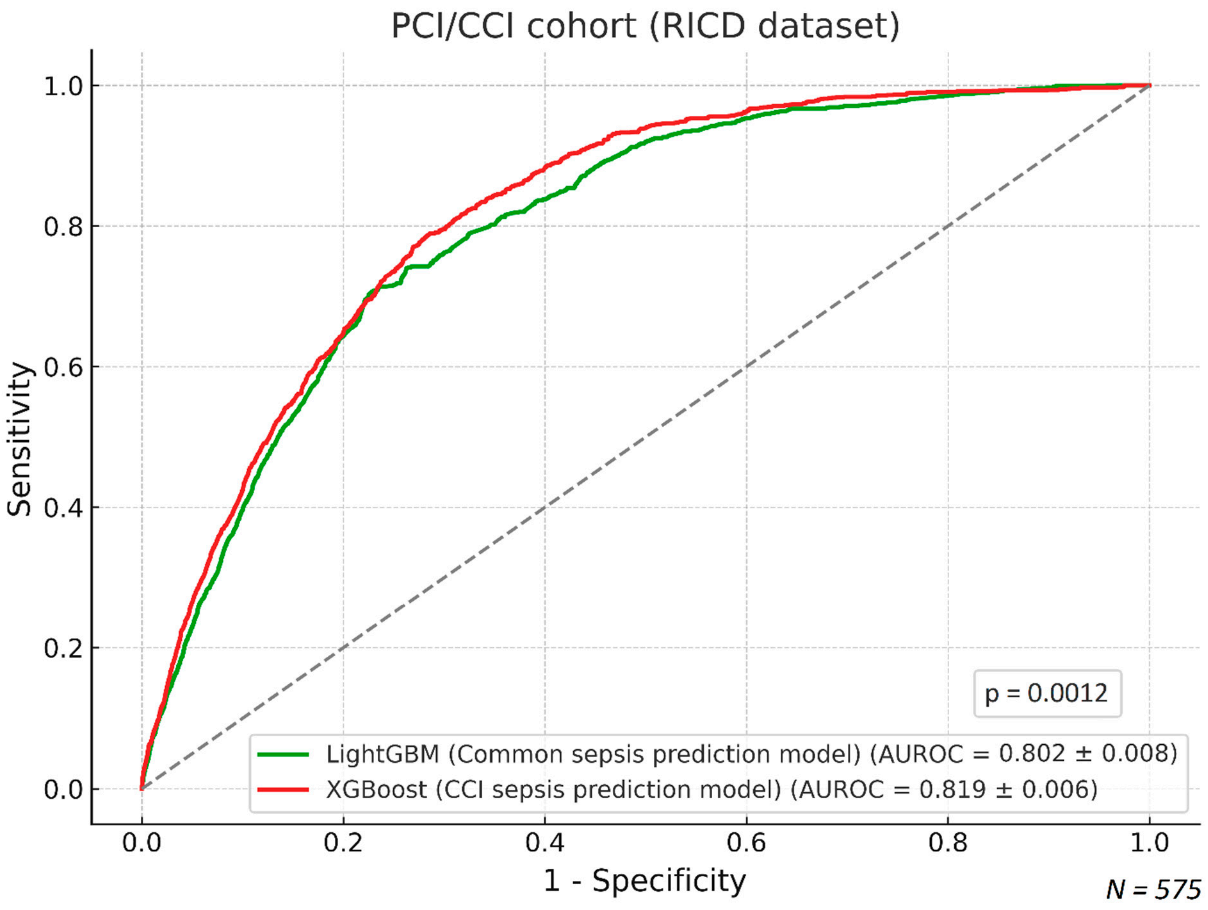

Abbreviations: PCI, prolonged critical illness; CCI, chronic critical illness.

Figure S4. Force plot illustrating predictor contributions for two patients from the RICD dataset.

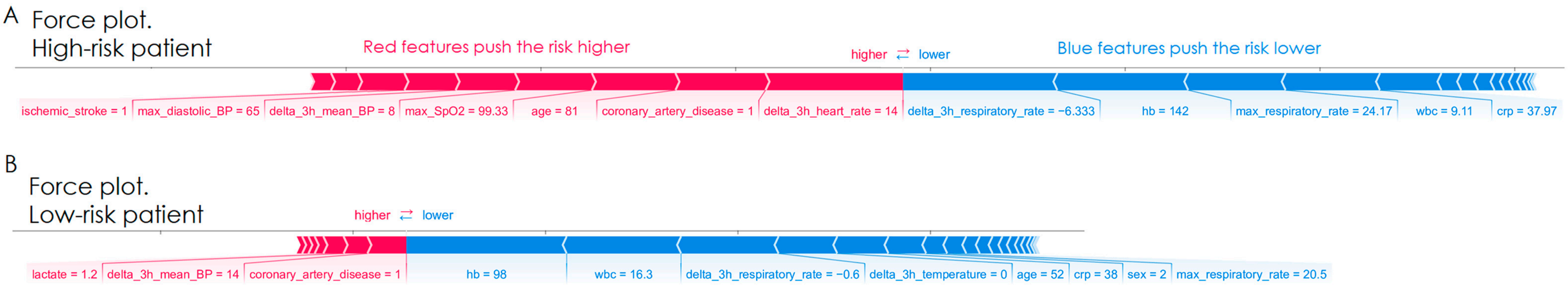

Force plot is a visualization based on SHAP (SHapley Additive exPlanations) values that illustrates how individual features contribute to the predicted risk for a specific patient. Red segments represent features that increase the predicted risk of sepsis, while blue segments indicate features that decrease it. The size of each segment reflects the magnitude of the contribution. In this figure, two patients from the RICD dataset are shown:

A – a patient with high predicted sepsis risk (RICD hospital ID 2249216, ICU hour 241),

B – a patient with low predicted risk (RICD hospital ID 4464602, ICU hour 1055).

Figure S5. Calibration curve for XGBoost model (train set).

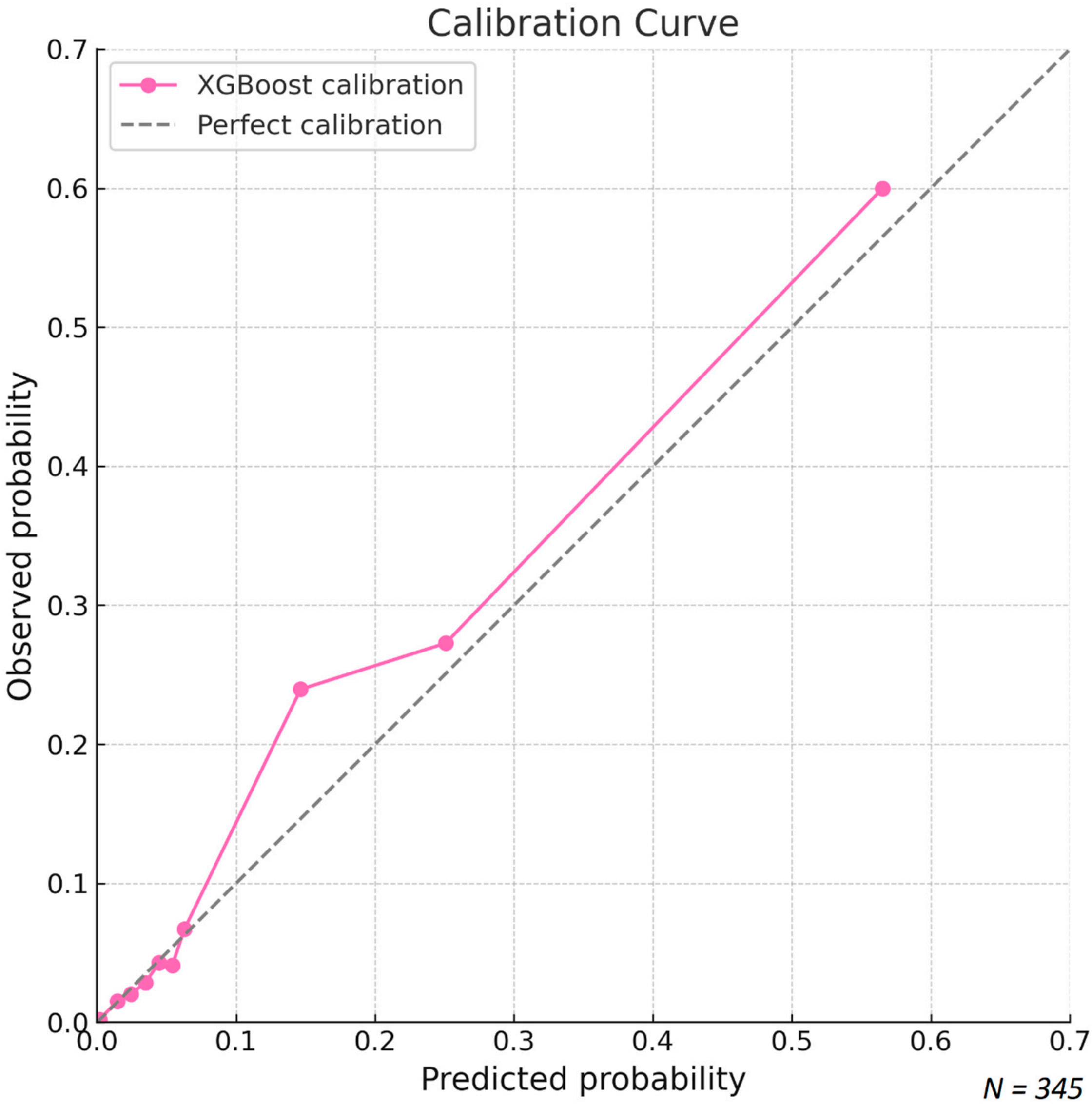

The calibration curve compares the predicted probabilities generated by the XGBoost model with the observed incidence of sepsis in the training dataset.

The diagonal dashed line represents perfect calibration, where predicted probabilities exactly match observed outcomes. The solid pink line illustrates the model's actual calibration performance. Overall, the model shows a tendency to underpredict the risk of sepsis in the mid-range probability bins (0.1–0.3), while demonstrating good calibration in the lower (<0.1) and higher (>0.4) probability ranges.

Figure S6. ROC curves of the XGBoost model for hyperinflammatory and hypoinflammatory sepsis phenotypes (RICD dataset).

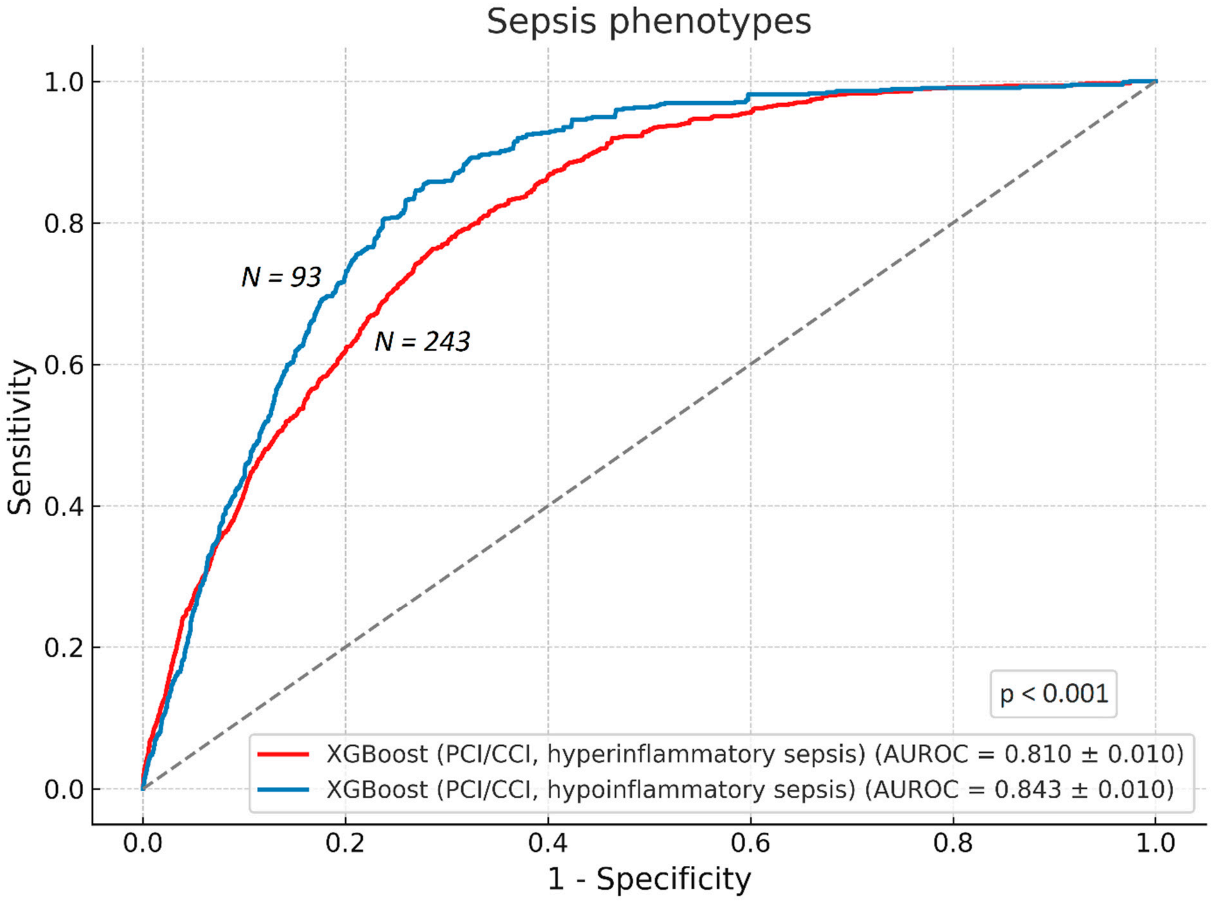

Figure S7. SHAP summary plots of the XGBoost model for hyperinflammatory and hypoinflammatory sepsis phenotypes (RICD dataset).

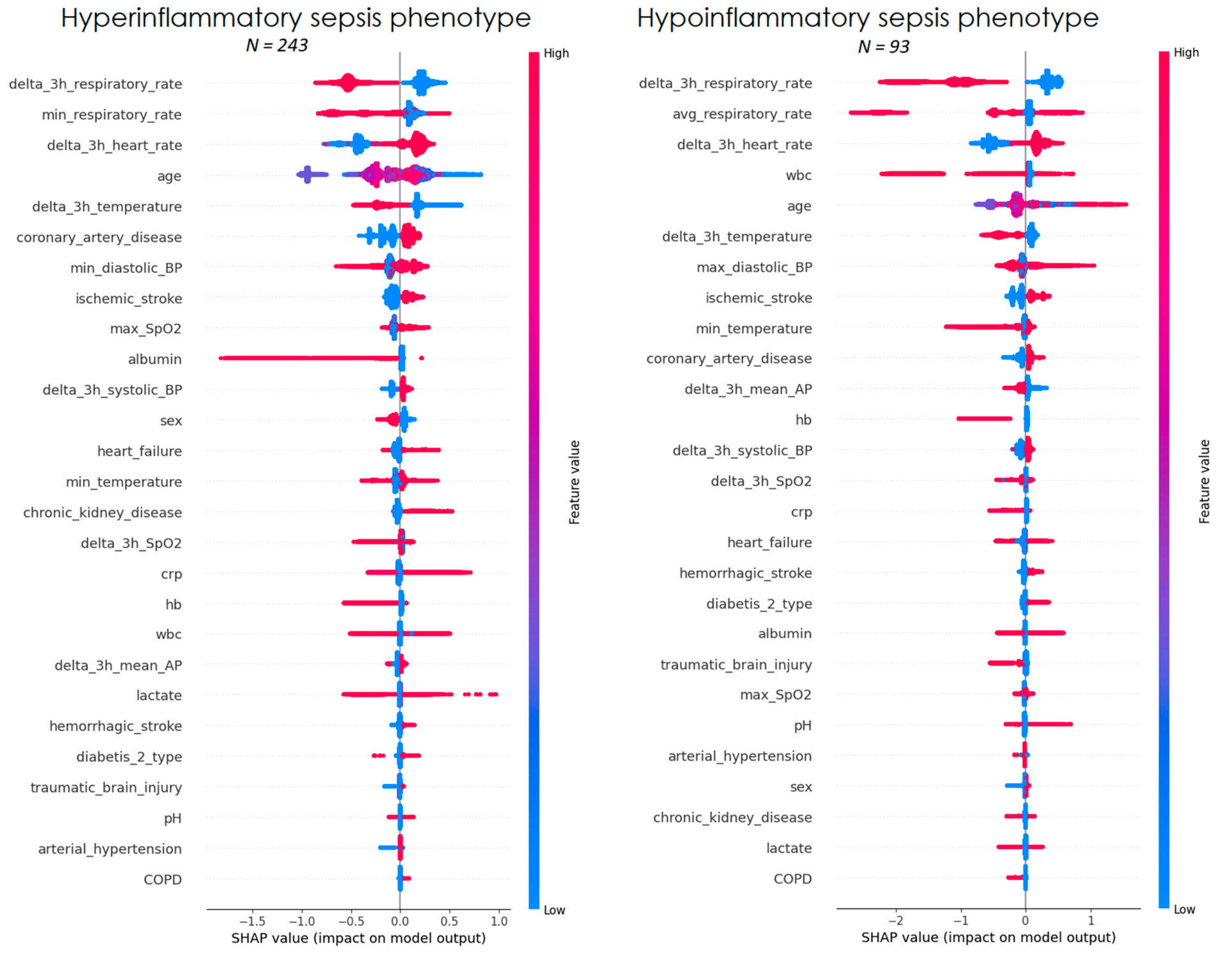

This figure shows SHAP (SHapley Additive exPlanations) summary plots, which illustrate the impact of individual predictors on the model output for two distinct sepsis phenotypes. Each dot represents a patient, with the color indicating the feature value (red = high, blue = low) and the horizontal axis showing the SHAP value, i.e., the contribution to the predicted risk.

For both phenotypes, respiratory rate and heart rate dynamics (e.g., delta_3h_respiratory_rate, delta_3h_heart_rate) show high importance, while the direction and magnitude of impact for other features differ between groups, indicating phenotype-specific patterns of predictor impact on sepsis risk estimation.

Figure S8. Decision curve analysis for the XGBoost model (RICD dataset, balanced 1:1 sample).

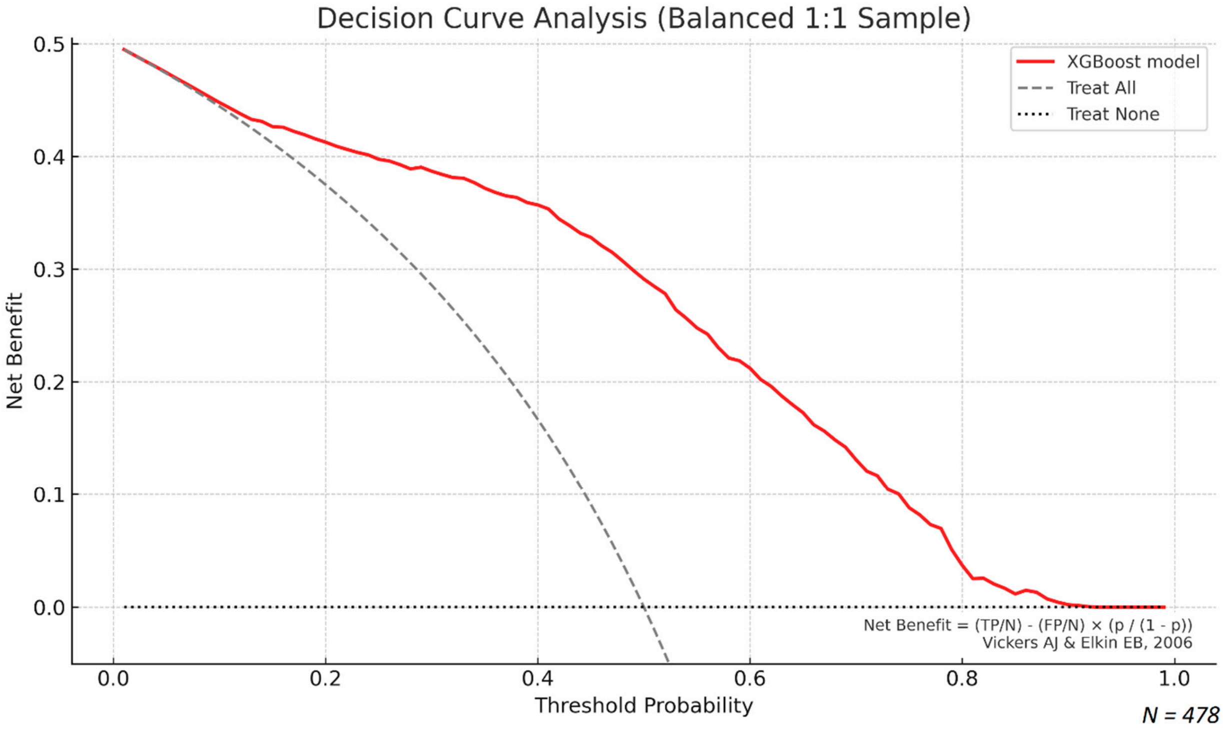

The decision curve analysis (DCA) evaluates the clinical utility of the XGBoost model across a range of threshold probabilities. The red line represents the net benefit of using the model to guide decisions, compared to two default strategies: treating all patients (dashed line) and treating none (dotted line). The model demonstrates a higher net benefit than either default strategy over a clinically relevant range of threshold probabilities, particularly between 0.4 and 0.7. This indicates that the model may improve decision-making by identifying patients most likely to benefit from early sepsis interventions.

A net benefit is calculated using the formula:

Net benefit = (TP/N) – (FP/N) × (p / (1 – p)), as described by Vickers & Elkin (2006).

Figure S9. Example of sepsis score dynamics over time predicted by the XGBoost model (RICD dataset, individual patient trajectory).

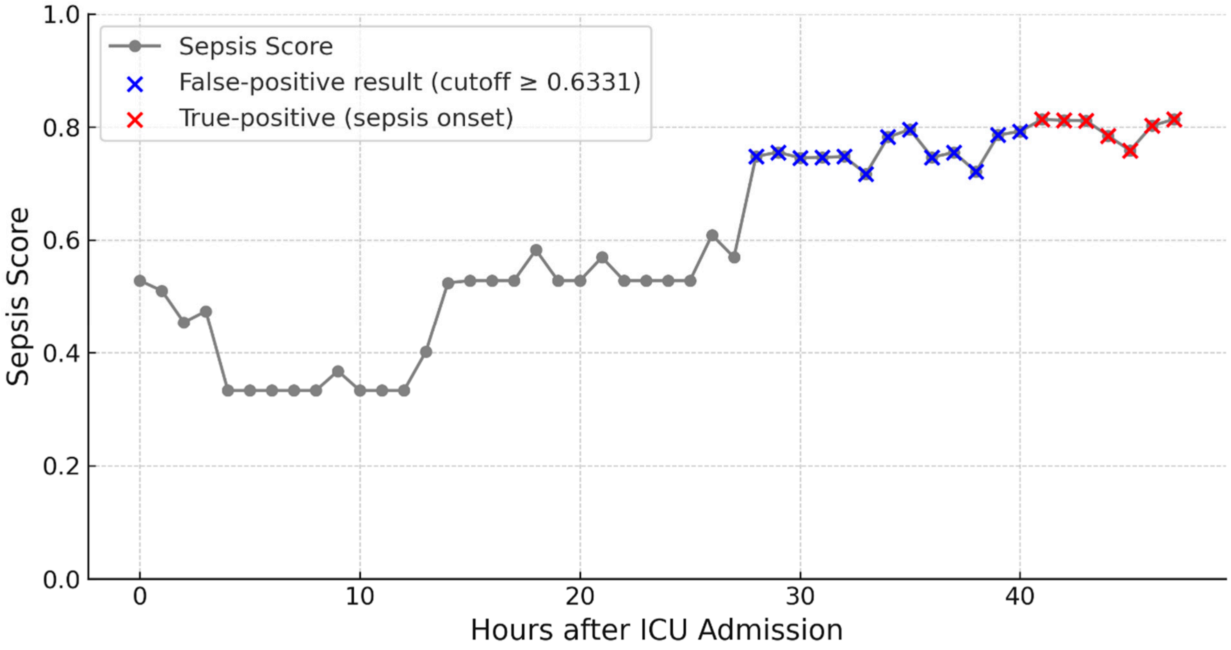

The figure illustrates the hourly sepsis score predicted by the model in relation to ICU stay (RICD hospital ID 4086934). Blue markers indicate false-positive results (cutoff ≥ 0.6331), red markers highlight true-positive predictions.
